# Micro-narratives: A Scalable Method for Eliciting Stories of People’s Lived Experience

**DOI:** 10.1145/3706598.3713999

**Published:** 2025-04-25

**Authors:** Amira Skeggs, Ashish Mehta, Valerie Yap, Seray B Ibrahim, Charla Rhodes, James J. Gross, Sean A. Munson, Predrag Klasnja, Amy Orben, Petr Slovak

**Affiliations:** MRC Cognition and Brain Sciences Unit, University of Cambridge, Cambridge, United Kingdom; Department of Psychology, Stanford University, Stanford, California, USA; MRC Cognition and Brain Sciences Unit, University of Cambridge, Cambridge, United Kingdom; Department of Informatics, King’s College London, London, United Kingdom; Department of Informatics, King’s College London, London, United Kingdom; Department of Psychology, Stanford University, Stanford, California, USA; Human Centered Design & Engineering, University of Washington, Seattle, Washington, USA; School of Information, University of Michigan, Ann Arbor, Michigan, USA; MRC Cognition and Brain Sciences Unit, University of Cambridge, Cambridge, United Kingdom; Department of Informatics, King’s College London, London, United Kingdom

**Keywords:** Human-AI collaboration, methodology, qualitative data collection

## Abstract

Engaging with people’s lived experiences is foundational for HCI research and design. This paper introduces a novel narrative elicitation method to empower people to easily articulate ‘micro-narratives’ emerging from their lived experiences, irrespective of their writing ability or background. Our approach aims to enable at-scale collection of rich, co-created datasets that highlight target populations’ voices with minimal participant burden, while precisely addressing specific research questions. To pilot this idea, and test its feasibility, we: (i) developed an AI-powered prototype, which leverages LLM-chaining to scaffold the cognitive steps necessary for users’ narrative articulation; (ii) deployed it in three mixed-methods studies involving over 380 users; and (iii) consulted with established academics as well as C-level staff at (inter)national non-profits to map out potential applications. Both qualitative and quantitative findings show the acceptability and promise of the micro-narrative method, while also identifying the ethical and safeguarding considerations necessary for any at-scale deployments.

## 1 Introduction

The ability to collect stories of participants’ lived experiences is crucial to understanding the challenges and opportunities associated with supporting individuals in managing their physical and mental health. There are many design contexts where HCI researchers might benefit from the ability to collect a large sample of users’ stories quickly and with limited effort (for both the participants and the research team). Imagine, for example, trying to understand the range of emotionally difficult experiences that young people struggle with online, or collecting the challenges that patients experience when receiving a behavioural change intervention in primary care across a large city or a state. Answering such questions likely presents several methodological challenges including (i) the potential for *high heterogeneity in responses*, especially across cultural, economic, and educational divides (i.e., a large dataset might be necessary before thematic saturation); and (ii) the difficulty of *reliably eliciting rich-enough qualitative information* (e.g., due to lack of time, motivation, or willingness to engage) from a sufficiently large proportion of target participants.

In other words, we so far lack approaches that could collect rich qualitative narratives from participants at scale (e.g., >150 participants) without undue participant burden and excessive costs of conducting the research: (i) interviews or detailed diary studies provide the necessary depth of understanding of participants’ experience, but are resource intensive and pose a substantial burden on the populations and the research team; (ii) approaches such as cultural probes are often bespoke and are similarly complicated to scale and resource (cf., [28]); and (iii) while questionnaires (including EMAs) can be deployed at scale, they often struggle to capture the depth of users’ emotional experiences (cf., [76]).

**Figure 1:**
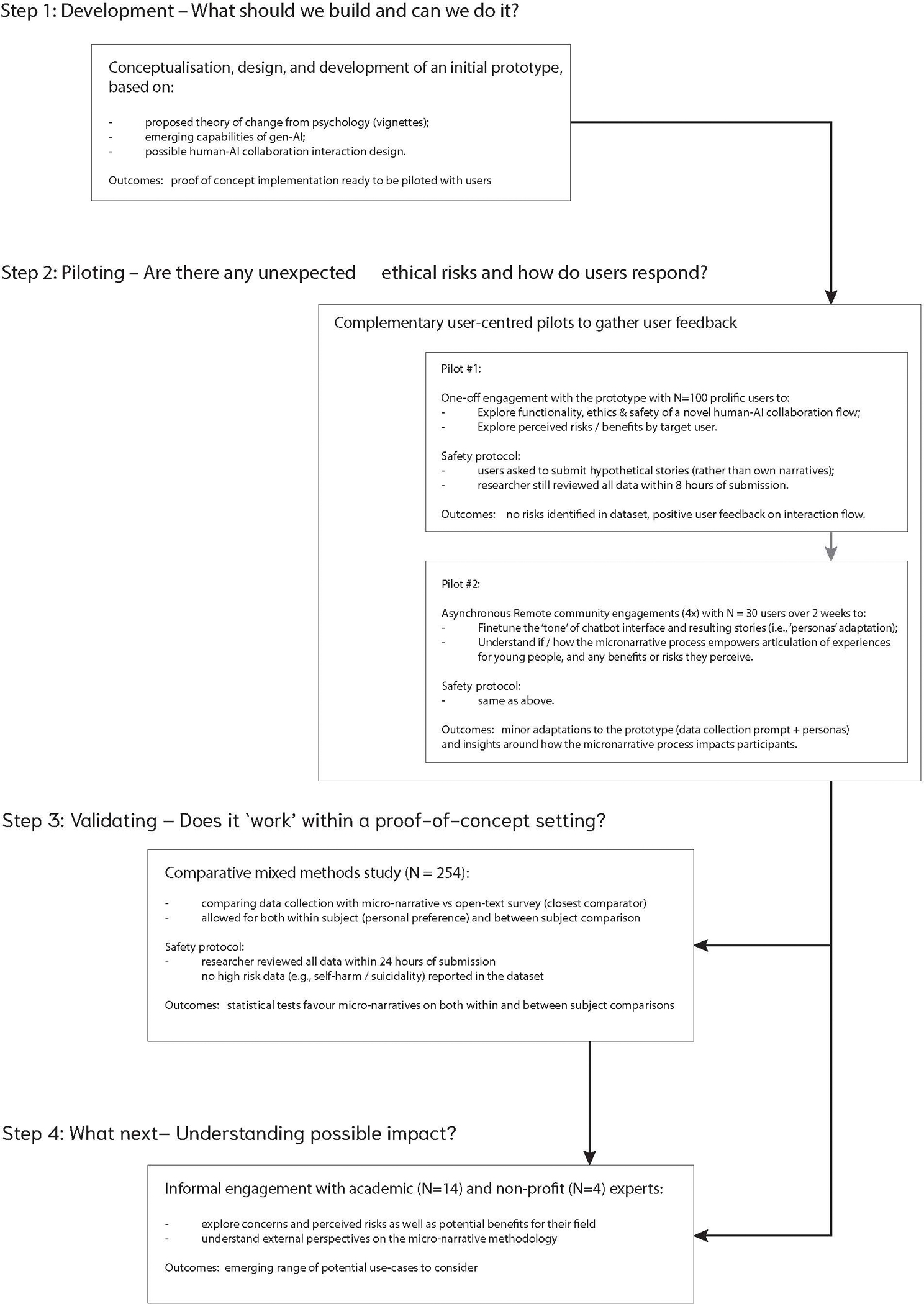
Overview of the research steps and their connections

This paper develops and tests a novel narrative elicitation method—based on human-centred AI interaction design—that could start to address these issues. We proceed in four steps, as illustrated in Figure 1: First, we outline the **design and implementation** of an AI-powered system to enable participants to articulate ‘micro–narratives’ of their personal experiences with only limited effort, regardless of their writing competency. We draw inspiration from prior research in psychology and HCI that uses researcher-generated vignettes to convey emotional experiences in ways that are succinct and understandable to participants (cf., [10, 17, 24, 31, 53, 70]). The proposed system aims to flip this approach: i.e., to explore the possibility of collecting *participant-generated* vignettes, as a way to enable them to articulate and share succinct-but-understandable stories about a specific aspect of their lives. The core design innovation involves scaffolding the user’s cognitive trajectory needed to develop a vignette-like narrative of their experience (cf., [67]), drawing on a new human-AI collaboration workflow (cf., [80]). Second, we **explore the feasibility and acceptability of a proof-of-concept prototype** in a series of 2 complementary pilot studies—an initial pilot (N=100) and a second pilot involving a 2-week asynchronous remote community (N=30)—with youth aged 18–20. To ensure a safe initial test of this novel human-AI flow, youth were asked to only submit ‘hypothetical’ stories rather than disclose any personal challenging experiences in the pilot studies. The aims were to gather user feedback on the proposed human-AI collaboration design, explore perceived ethical risks and potential benefits as seen by our target population, and guide design changes as suggested by youth. Third, we **validate the resulting adapted micro-narrative elicitation tool** in an online comparative study (N=254 youth, aged 18–20) that compared the prototype with an analogously worded open-ended survey question (as the closest comparator). The experimental design allowed us to compare both between-subject and within-subject effects. Finally, we **map out a range of potential use-cases** for micro-narrative tools, based on informal discussions with 4 C-level staff at major national and international non-profits, as well as 14 established researchers (median citations 13.1k) across a range of disciplines, including clinical psychology, behavioural health, communication studies, implementation science, and HCI.

Our findings suggest that the micro-narrative elicitation tool could be feasible to deploy and seems acceptable to both our case-study participants as well as academic/non-profit experts. In our pilot studies, participants responded positively to the step-by-step process of developing a vignette-based story with some describing how the system helped them to ‘make sense’ of their experiences and facilitated personal reflection and understanding. When probed about potential risks, and how the interaction could be improved, participants raised concerns about data privacy and the ability of AI systems to handle sensitive personal experiences. The data from the comparative study shows that youth saw micro-narrative elicitation as preferable to traditional open-text survey methods on a range of factors: For example, *between-subject comparison* found that the micro-narrative process was reported as significantly more helpful (than the open text form) for articulating the experience, less difficult to respond to, more accurate for capturing the objective features of the situation and that others who read the final product would understand participants’ experiences better. The *within-subject comparison*^1^ found that participants were 4.5x more likely to report that micro-narrative was easier to capture their experience, 2.8x more likely that it was more appropriate for (other) youth, 6x more likely that it is more helpful for making sense of the experience, and 4.8x more likely to better support them in thinking about how to address their social media challenges. Finally, the expert engagements showed a similarly positive response. For example, all four non-profits and seven researchers are currently working with us to incorporate the narrative elicitation tool into their work.

While these initial results are promising, many ethical, epistemological, and pragmatic questions remain before this method can be deployed within the community at scale. We discuss the future research necessary to determine the context-specific safeguarding protocols that would be necessary, the impact that such narrative articulation could have on users, as well as open questions about how best to analyse the resulting data (which is, in effect, co-created between a human and the AI-support tool). To encourage open-source development and research on these critical questions, we describe the full system design—including all LLM prompts—in Appendix A; and will make the system source code publicly available under a Creative Commons license at the point of publication. The code will be further modularized to ease further adaptation and extensions by other research teams.

## 2 Related Work

The design challenges raised in the introduction are connected to multiple areas of research, which we review below: We start by outlining the amazing breadth of data collection approaches employed within HCI in the context of collecting participants’ lived experiences, with a specific focus on how these balance the trade-offs between qualitative richness in the collected datasets and the participant/researcher effort this richness requires. We then briefly provide an overview of the psychology research around vignettes, which served as inspiration for our approach – i.e., seeing vignettes as a potentially useful form of ‘template-driven storytelling’. Finally, we review the recent work in HCI that explored the use of Large Language Models (LLMs) to support digitally mediated data collection as well as narrative (or reflection-oriented) support.

### 2.1 HCI methods to elicit / collect participant experiences

Deeply engaging with the lived experiences of stakeholders is a fundamental concern for human computer interaction (HCI) research, with so much excellent methodological and conceptual scholarship published over the last decades that we are unable to fully cover it here (cf., [7, 26, 59, 66]). In the context of this paper, therefore, we specifically focus on the ways in which most commonly used existing methods—such as interviews, diary studies, probes, questionnaires, and ecological momentary assessments (EMA)—*trade-off striving for qualitative richness of the collected datasets, and the required effort from the researchers / participants to do so*.

For example, interviews require substantial researcher and participant effort to collect data (including scheduling coordination, and the time spent talking), in addition to the non-trivial analytical resources required to make sense of the collected data – but result in a deep, nuanced understanding of participants’ perspectives. Similarly, ethnographic methods might utilise a combination of interviews and long-term participatory observations to seek even more granular understandings about people’s beliefs, lived and felt lives [79], as well as in-depth analysis of social practice within their communities; which further increases the resources required from the researcher within data collection & analysis. Diary studies and other probe-based approaches^2^ are a related set of qualitative methodologies which seek to address the challenges of developing a longer-term understanding of participants’ lived experience. The difference in approach is that these methods do so by deploying digital or physical data-collection instruments directly into participants’ daily lives. In the context of our argument here, such methods are thus transferring some of the data collection burden onto the participants (e.g., requiring a daily diary entry, or a thoughtful engagement with a cultural probe) – albeit often in playful and insight-provoking ways – and are intended to inspire ideas and prompt a deeper dialogue between researchers and participants [20, 21, 27]. Finally, methods such as questionnaires and more recently ecological momentary assessments are traditionally built with a different goal in mind: a large-scale collection of mostly quantitative information, often in the form of pre-determined, multiple-choice questions, often in response to the known challenges of reliably collecting open-ended qualitative insights (cf,. [81]). In these cases, the trade-offs tilt towards low-burden (for researchers and, hopefully, participants) which then enables large-scale data collection, but has the side effect of reducing the qualitative richness of information that can be collected.

We note that none of the qualitative methods outlined above (interviews, ethnographies, diary studies, or probes) are traditionally used to collect data from hundreds of participants (or more) in HCI (cf., [8]) – these methods have not been practically or epistemologically designed to answer questions requiring such high sample sizes, at least not for short term, iterative studies. It is, however, not uncommon for multi-year projects in sociology or anthropology to require in-depth interviews with hundreds of participants when complex social questions are explored (cf., for influential examples relevant to HCI [29, 41, 77]) – but the time, financial, and human resource costs necessary for the data collection *and* analysis within such projects are immense.

In summary, the existing approaches do not provide an easily accessible set of methodological tools that would help address the type of questions outlined in the introduction. In other words, at the moment, if one needs to collect qualitatively rich stories from a large number of participants, the existing methods seem to be associated with high burden and cost for either the researchers or participants (and most commonly both) – even if there are examples of research questions for which such a price is worth paying.

### 2.2 Vignettes as examples of the usefulness of short narratives in psychology research

In preparation for the conceptual system design in Section 3, we now turn our attention to ‘vignette’ studies. This methodology, initially emerging from ethnographic research, uses *vignettes*—i.e., short scenarios—as a way of providing participants with succinct and widely understandable representations of a situation of interest, which the participants then react to. For the purpose of this paper, our interest in vignettes is not in how they are currently used—as a prompt to elicit a participant response—but rather in what these studies implicitly show about the vignette format *as a ‘storytelling device’*: that is, their apparent ability to convey potentially intricate and emotional stories within a short and template-like ‘micro-narrative’.

#### Vignettes as qualitative response elicitation.

Much of research in social sciences has focused on using vignettes as ‘inputs’ into a qualitative interview process in social research, helping the researchers unpack topics that might be otherwise difficult to engage with and struggle with social desirability bias. In this sense, the short ‘descriptive scenarios’ can effectively elicit responses to sensitive topics, and enable the participants to focus on elements that are particularly important for the researchers’ topics of interest – with prior use both within sense-making work as well as intervention development (cf., [71]).

In these instances, the researchers place importance on creating believable and ‘realistic’ vignettes to reduce the tendency of participants to answer in general / abstracted / hypothetical terms. For example, Sampson et al [60] articulate the value of their use of ‘real-life’ (i.e., directly based on field observations / interviews) with the following quote:

Overall contribution of ‘real-life’ vignettes to the outcomes of studies A and B, we consider that their greatest impact was in encouraging participants to engage with the materials presented to them to such an extent that interviewers were temporarily granted insider status within their ‘communities of practice’ [37]. Here the vignettes: stimulated engagement and openness; reduced the tendency for idealised answers; facilitated the development of a high degree of trust in situations where participants were suspicious; and generated credibility. This allowed participants to discuss matters that would generally be off-limits. In this context, they were able to reveal the ‘unacceptable’ (errors, deviant/prohibited practice, non-masculine behaviour) and reflect on the proscribed.

The use of vignettes in similar contexts is thus exploratory and/or interpretative: aiming to help elicit in-depth qualitative responses from participants to better understand the ‘tacit’ knowledge and social practice that might be otherwise difficult to uncover; whilst offering participants carefully scoped ‘microcosms’ (cf., [69, p. 343]) to react to as a way to lower barriers to open communication.

#### Vignettes as an experimental research tool.

The second way in which vignettes are used in research is as tools in experimental research. Such an approach is commonly applied in experimental psychology research, such as fields examining decision-making, moral judgments, cognitive psychology, and increasingly HCI^3^. In these instances, the focus is on uncovering the generalisable cognitive patterns (or their variance across populations). The (sets of) vignettes are seen as providing a stable and easily understandable set of stimuli, which enable the researcher to manipulate and examine the impact of varying parameters of importance. For example, [64] describes using such an approach to understand the inequalities derived from ‘unwarranted variations’ in health care — such as those hypothesised due to implicit biases (e.g., delays in cancer or poorer reported experiences with doctors for patients of marginalised backgrounds).

In these contexts, sets of vignettes are created to share a common structure while allowing for variation in elements that are assumed to have theoretical importance. In this instance, vignettes are described as:

short, carefully constructed depiction of a person, object, or situation, “representing a systematic combination of characteristics” [. . . ] In experimental vignette studies, vignettes are used to explore participants’ attitudes, judgements, beliefs, emotions, knowledge or likely behaviours by presenting a series of hypothetical yet realistic scenarios across which key variables have been intentionally modified whilst the remaining content of the vignette is kept constant. Such studies seek to generate inferences about cause-and-effect relationships by considering the nature of each vignette, and participants’ subsequent responses to these vignettes. [64]

We note that vignettes are still serving a communicative role in these studies and are often designed to convey / elicit emotions. However, in contrast to the qualitative studies, more focus is given to a theory-informed template-like form of the vignette. Interestingly, this then allows individual aspects of the vignette-described story to be independently manipulated while retaining story coherence – an observation that we will use in our design in section 3.

### 2.3 Digitally-mediated data collection and narrative/reflection support

Finally, there has been an explosion of interest in recent years in understanding the range of human-AI collaboration tasks, especially as enabled by the rise of generative AI and Large Language Models. To the best of our knowledge, no research to date directly addresses the type of narrative articulation support that is of interest in this paper. The closest related work can be identified across two streams of prior research: the first is exploring the support of *participants’ reflection or articulation*, which is to date mostly centred under the banner of creativity; the second is the focus on *streamlining data collection* techniques. We review the most relevant work from each of these streams below.

#### Reflection & articulation.

Much recent work has focused on the role that LLM systems can play in supporting a spectrum of human-AI co-creation tasks (cf., [46]). For example, Luminate [40] is designed to help users explore and navigate a wide range of possible ideas in the context of supporting professional writers. The LLM components of the system are used to (i) generate possible categories; and then (ii) allow the user control and exploration of the range of options with a ‘dimension-guided’ response generation. Similarly, CharacterMeet uses LLMs to support creative writers in story writing through conversations with LLM-driven story characters [55]. As a further example of using LLM systems for creative tasks, the ‘Idea Machine’ was developed to facilitate idea generation and reflection across a range of topics, enabling users to expand upon, rewrite and connect ideas [16]. Research has also examined the use of LLMs to edit written documents, demonstrating how these systems can effectively address both grammatical and content changes across a wide range of editing tasks [34]. Recent studies in education have also considered the potential of LLMs to provide personalised feedback on students’ written work, finding that these tools enhance students’ writing proficiency and motivation [48, 54]. Across all of these examples, the research suggests that there is an opportunity for LLMs to empower human creativity, including articulation of narratives – while still enabling the user to remain in charge and keep control over the final outputs.

#### Streamlining data collection – surveys.

Recent research started exploring the potential of AI-powered interactions as an alternative form of data collection from users. For example, Xiao et al. [81] investigated the potential of pre-GPT chatbot systems to conduct conversational surveys with open-ended questions, in the case study context of game market research. The key motivation was to reduce survey fatigue and user burden, to reduce the likelihood that users will skip such questions or provide low-quality responses. Results from their experimental study showed how the chatbot-driven surveys had substantially higher completion rates, and somewhat higher informativeness and relevance (with much fewer ‘gibberish’ answers), with participants volunteering more detail and engaging for longer. These findings were motivated by prior research suggesting that engaging chatbot systems as data collection tools may improve response quality, participant engagement, and enjoyment relative to traditional survey methods [1, 32]. These chatbot systems may be further enhanced by humanization techniques, which improve respondents’ perceptions of the chatbot and increase interaction time [58].

#### Streamlining data collection – narratives.

In the last months, several research projects have extended such an approach from survey open-ended questions to explore the potential of Large Language Models in collecting longer form qualitative data, often in the form of a chatbot-led ‘interview’. Wei et al. [76] developed a set of GPT-3 powered chatbots to “collect user self-reports while carrying naturalistic conversations”, motivated by the challenges with tracking burden for participants if such details were to be captured manually. Focused on four health contexts (sleep, food, work productivity, and exercise), the authors showed how carefully prompt-engineered bots were able to collect data on pre-determined aspects of the context in question (e.g., food intake for each of the main meals of the day), although not in an entirely reliable way. The authors also highlight the ethical & design challenges of potential problematic responses – cf., the wider discussion on LLM-alignment [18].

In more clinically sensitive work, Kim [33] developed a “MindfulDiary” to support psychiatric patients’ journaling experiences, with the interaction driven by AI-generated prompts and reflective questions (driven by OpenAI’s GPT4). The authors’ aim was to reduce the burden that traditional daily diary entries would impose on patients, as well as enhance self-exploration and aid in expressing their experiences and emotions. The resulting diary interactions were well received by patients and therapists, with the system helping to mitigate some of the challenges associated with traditional journalling approaches: resulting in interactions that “ensured the users are not overwhelmed by the task, and guided in documenting their feelings and experiences more richly.” Finally, Seo et al [62] developed an LLM-powered system to empower children to share and reflect on their emotional experiences. The short-term lab testing with 20 children showed promising results, with children being receptive to using ChaCha for disclosing emotions and stories – even, concerningly, those that they have never shared with their parents.

These systems show the promise of LLMs to help reduce participant burden and enable innovative approaches to supporting data collection, including the focus on supporting users’ narrative creation. However, most of these examples still shared the challenge of building reliable and robust systems based on prompt engineering inherently stochastic Large Language Models (cf., also [84]) and were often designed for highly specialised use contexts.

## 3 Step 1: Conceptualisation, design, and development of initial prototype

We framed our design aims to explore the opportunities for digitally mediated support that would, at the same time, provide: (1) enough *open-endedness*, to capture participants’ lived experience, in their words; (2) enough *consistency* in the set of core aspects covered within the stories, so that specific research questions can be addressed; while (3) *reducing the burden* for participants, so they remain willing to create and share their narratives, and ideally find value for themselves in doing so. Drawing on prior work on the user burden associated with remote open-text data collection [49, 81], we assumed that the key design innovation will need to be in reducing the associated friction and cognitive difficulty inherent to text-based narrative creation, especially for marginalised / at-risk populations (cf., [22, 33, 62]). As such, we were interested in exploring how the system could reduce user burden by including some form of scaffolding for the articulation process (cf., Section 2.3).

### 3.1 Design approach

The core insight driving our design answer to this dilemma of openness vs consistency vs burden was the way in which vignettes—as a narrative structure—seem to support a form of succinct-but-understandable storytelling. As we outlined earlier in Section 2.2, many vignettes in prior work can be seen as instantiations of particular ‘templates’ that provide focus for the resulting story: these do not constrain the content of the situation being described, but guide the author by specifying a set of aspects of the situation that should be included.

#### Design assumptions.

Based on the considerations above, we have articulated three design assumptions, that drove both our development work (described below) as well as the empirical studies (described in the next sections). We outline these below:

DA-1 The first design assumption we wanted to explore was whether supporting articulation of a story might consist of *collecting a set of carefully selected ‘fragments’ of the situation* (which are individually easy to answer), but which can be then *combined into a coherent narrative* (based on an underlying template) and serve as a helpful starting point for further adaptation if needed.
DA-2 Our second design assumption was that *this process could be supported through Large-Language Models*, targeting the steps in the cognitive process above that might be particularly burdensome on the user. In particular, we wanted to explore the potential of ‘agentic’ human-AI workflows: i.e., approaches where the target task is broken down into individual components, where ‘specialised’ LLM components are used to support individual steps of tasks, and at least some of the programme execution is governed by LLM outputs.
DA-3 Finally, our third design assumption was that, if we are successful, such an articulation support process will be perceived by the participants as:
  - *Simple and efficient*: e.g., easier than composing a narrative from scratch, such as in an open-text survey;
  - *Empowering and affirming*: enabling articulation of an experience in ways that feel to be “the user’s own voice” even if the user received LLM support; and potentially also
  - *Personally helpful / insightful* – e.g., that the process of articulation might lead to new insight / perspective on the reported experience.

In summary, the first assumption aimed to describe the ‘theory-of-change’ that would underpin the proposed new capability (cf., [67]) – i.e., guide how and why we would believe the interaction design would lead to the intended outcome. The second assumption highlighted the technical innovation which we assumed could not only reduce user burden but actively enhance their ability to express themselves (the novelty of which could explain why a similar design has not yet been proposed). Finally, the third assumption then focused on the user-implications of the resulting process – each aligned with the goals of reducing user burden, facilitating open-ended self-reflection, and increasing perceived benefit.

### 3.2 Proof-of-concept development

Two complementary design frameworks drove our design exploration and attempts to test the assumptions above.

#### Designing for cognitive trajectories.

First, we draw on Slovak & Munson’s framework [67] and its focus on clearly articulating the ‘cognitive trajectory’ that the design is aiming to support. In this case, we considered how the mental process of accomplishing this task might look for the participants if they were to be asked to articulate similar template-based narratives without any AI-support; and, most importantly, *which of the steps in such a flow might be most difficult and/or burdensome*. For example, a plausible expectation^4^ would be that participants might be first asked to *consider responses* to the *individual questions* within the template, then *reflect on the connections* between answers, and finally *combine and rewrite* their answers into a narrative that incorporates all of the information (potentially with adding further detail or editing previous answers). Our preliminary assumption was that the most difficult steps would likely be the *combine and rewrite stage*, especially if the proposed template is not closely aligned with a mental model that participants would have.

#### LLM-chaining as ‘crowdsourcing’.

Second, we were inspired by the recent approaches based on LLM-chaining as an analogy to prior crowdsourced workflows [80]. In our view, this appeared to be a promising direction that would enable the need to maintain a fixed (state-based) trajectory of the cognitive flow, with the openness and flexibility of LLMs for individual steps (cf., [33, 62, 65, 76]). In addition, such an approach allowed us to ‘enforce’ the assumed theory-of-change (i.e., the cognitive trajectory that we aimed to support), while the identified human/AI subtasks encapsulate the AI prompting into independent and easier-to-debug chunks [80]). Finally, we also assumed that such infrastructure adds useful modularity to the resulting human-AI workflow (cf., [65, 84]), e.g., allowing the inclusion of targeted safeguarding components (such as detection of self-harm / suicidality risk) into the workflow without the need to adapt other steps.

Across all of these, our implementation was based on prompt engineering literature (e.g., [78]) as well as our prior expertise with similar prompt engineering and design projects. We selected the underlying frameworks (langchain & streamlit) to enable rapid exploration and change, with the view that this will enable deeper co-production and active engagement from our youth participants (as well as experts) – which we assumed will be a necessary component given the focus on developing a new human-AI cognitive workflow, without clear prior literature on either the expected human-AI distribution of ‘tasks’ in support of story articulation or an understanding of user preferences.

#### 3.2.1 Proposed Human-AI workflow.

**Figure 2:**
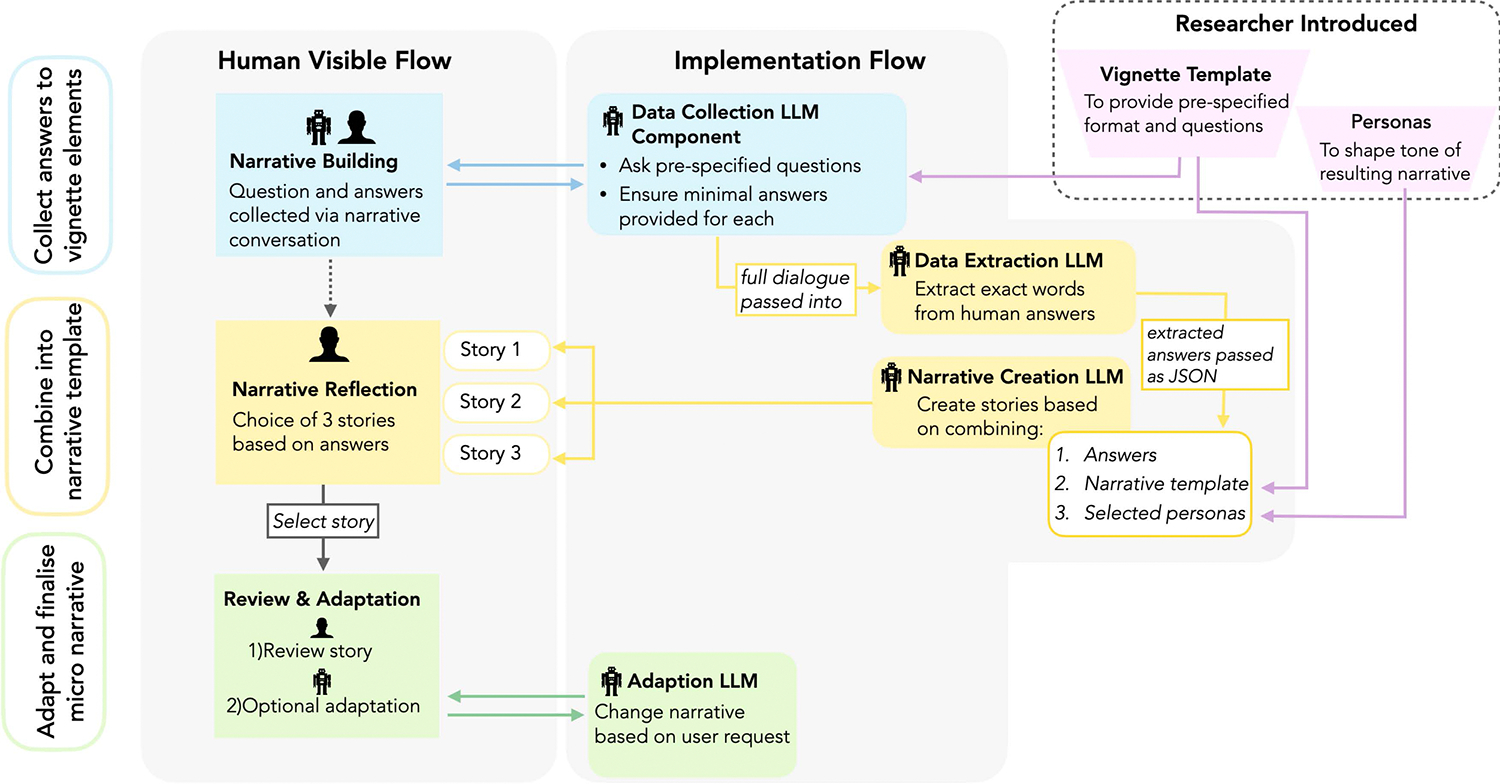
Overview of the three-stage human AI workflow

The envisioned workflow consisted of three stages described below and visualised in Figure 2. The aim was to mimic the cognitive flow outlined above and retain participants’ ability to share their stories in their own words (‘voice’) as much as possible while reducing likely friction through LLM support.

- *Stage 1 – Data collection:* Human-AI collaboration to collect answers to the selected questions (i.e., story ‘fragments’ that form the elements of the vignette) through a conversational interface. The LLM is prompt engineered to ensure that the participant is asked—and provides answers—to all questions specified in the vignette template through natural language conversation (cf., [62, 76]).
- *Stage 2 – Synthesis and narrative building:* Once the questions are answered, an AI-only component extracts the exact answers that users provided, and then combines these into a proposed narrative following the vignette template that (i) leads to a coherent narrative; (ii) directly incorporates what users’ said in their own words; while (iii) attempting to add as little additional information as possible. This narrative-building step creates three different versions of the narrative—implemented as ‘personas’—which should differ only in the tone of the ‘connective text’ that the LLM inserts when connecting the user’s answers to the ‘fragments’ into a coherent story.
- *Stage 3 – Review and selection:* Finally, users review the three versions of their story, and are asked to see if any of these 1) resonates with the ‘content’ of what they wanted to express and 2) feels like ‘their own’ voice (Human-only choice). We chose to provide three different ‘tones’ of the story to encourage a feeling of meaningful choice over which narrative feels best like ‘their own’ and also highlight the range of ways in which their words can be combined. The participants also have the opportunity to further adapt the story as needed, e.g., if the LLM workflow misrepresented any information, or did not include something the participants find crucial to their experience (Human-AI collaboration).

#### 3.2.2 Initial case-study context and implementation details.

As the initial case study, we focused on collecting young people’s narratives of challenging experiences on social media. In particular, we hoped to elicit detailed stories of what has happened online for them, and how they then interpreted/reacted to these situations. We note that this is a surprisingly under-studied question in the social communication field: despite the extensive research examining social media’s well-being impacts, the specific social media challenges impacting young people’s well-being remain poorly understood (cf., [35, 72]). Moreover, within social media research, there continues to be a lack of qualitative research that considers young people’s lived, individual experiences to answer questions about social media’s well-being harms (see also [30, 61, 77]).

Further details of how we developed the prototype for this proof-of-concept case study are described in Appendix A, including the full prompts used. The source code will be published on github under a Creative Commons Licence and made freely available upon publication; for the review process, we attached the source code as a supplementary file on PCS.

## 4 Step 2: User-centred pilots

Our next aim was to start exploring the ethical risks of the micro-narrative method and how users responded to it as a data collection tool within the selected case study domain of understanding youth social-media challenges. Methodologically, we approached these initial studies as a combination of technology probes & prototype pilots – our aims were to provide an initial exploration of the perceived acceptability, reliability, and any challenges of the method from our target population (Pilot study #1), as well as enable young people to engage with and suggest changes to the presented prototypes through more in-depth qualitative methods (Pilot study #2). Both studies were approved by the institutional ethics committee at Kings College London, and reflected two key design decisions:

- The first was related to *Responsible AI design and safeguarding* concerns. In particular, we chose to instruct the users to engage with the system by reporting hypothetical scenarios (that could be informed by their experiences), but not to directly share their personal social media struggles. Importantly, the system (and broader study) was also presented to participants as a *data collection tool*, where we stated that the aim of the prototype was to collect data about youth’s social media experiences and designed the chatbot’s hard-coded initial message to clearly reflect this purpose. We believed this precaution to be necessary as we could not fully predict how the LLM system would react to users’ emotional experiences or whether users would find the conversations jarring, and wanted to avoid instances where personally meaningful experiences could potentially be re-described by the model in an insensitive or triggering manner (i.e., concern for participant safety and opportunity to test our safeguarding protocol in a safer context).
- The second design decision focused on *utilising remote data collection platforms* (specifically Prolific). Our aim was to test the prototypes with a sample of young people across the UK, with a specific interest in reaching those who would not be able to travel for an in-person meeting in a central London location. We also assumed that such remote collection mimics the most likely application of the method and built on prior examples of successful remote user-centred design approaches (cf., [19, 45]).

In what follows, we describe the high-level methodology and key outcomes from these two studies.

### 4.1 Pilot Study #1 – one-off online engagement

Pilot #1 aimed to explore key ethical and safety considerations of the micro-narrative method as a data collection process. The study was conducted via Prolific and consisted of three components: demographics and background questions (Qualtrics), prototype interaction (Streamlit), and feedback survey (Qualtrics), which captured both quantitative and qualitative data about the interaction experience. The prototype interaction is shown in Figure 5. In total, N=100 participants were recruited from Prolific and compensated £10/hour via the recruitment platform (averaging £3.30 for the 20-minute study). All protocols are included in the supplemental materials. Our safeguarding protocol ensured that all data was seen by a research psychologist within 2 hours of submission. In cases of concerning disclosures or clear evidence of distress during the interaction, researchers with safeguarding training would contact the participant and provide additional mental health resources (no such cases occurred in our pilot studies).

#### 4.1.1 Data analysis.

**Figure 4:**
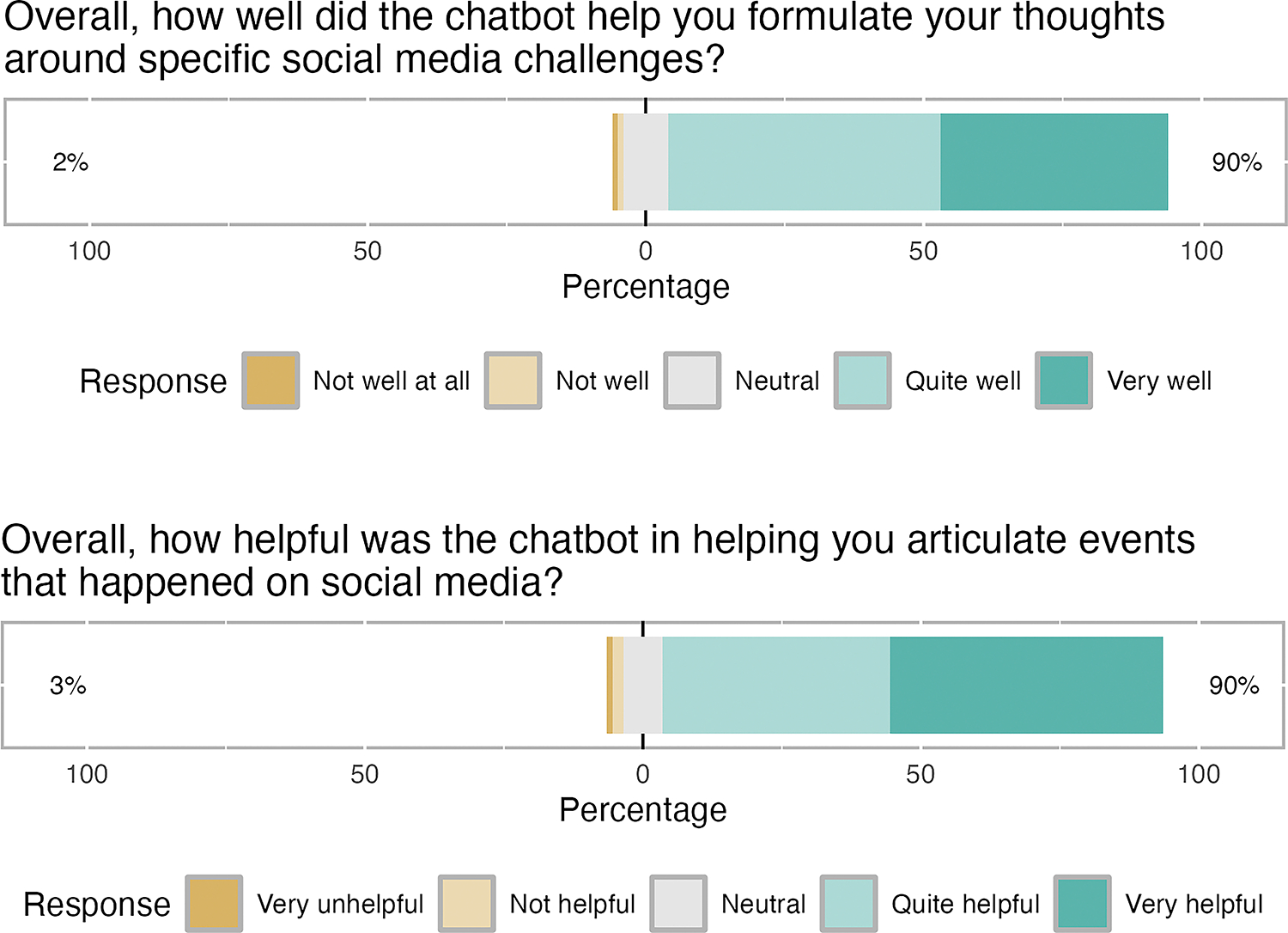
Quantitative feedback for acceptability of the prototype in Pilot #1.

**Figure 5:**
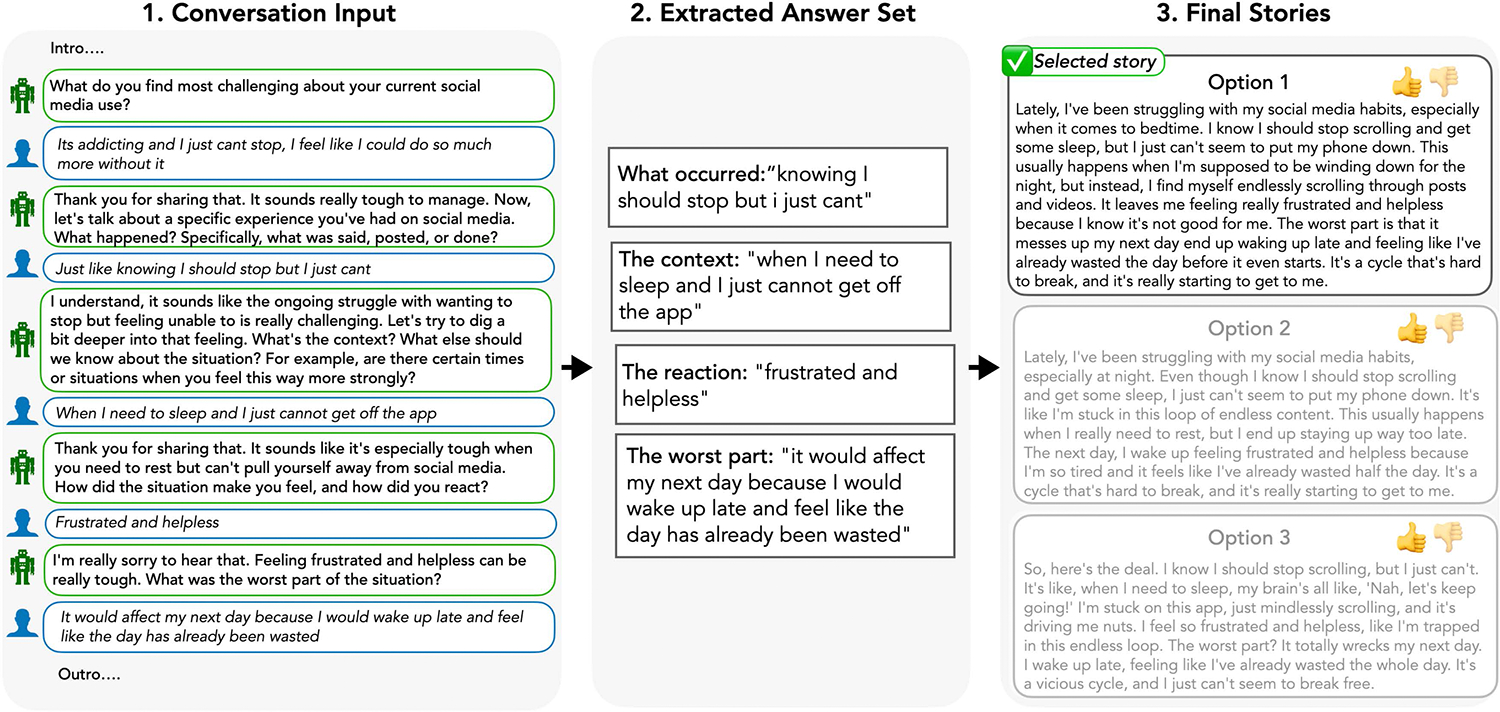
Example prototype interaction from Pilot #1

We conducted *three streams of analysis* for Pilot #1. The first focused on the performance of the LLM model and aimed to understand whether the data collection tool was collecting the intended data and accurately representing participants’ experiences in the micro-narrative output. To check this, the first author analysed individual participant interactions with the prototype (as JSON files exported from Streamlit). Participants’ raw answers were compared against the final LLM-generated scenario to evaluate whether the model was accurately extracting data from participants’ input, with Authors 1 and 10 meeting regularly throughout the data collection process to discuss the results. We also read the interactions and scenarios to check for model hallucinations or deviations from the scoped interaction flow. The second stream of analysis was a descriptive analysis of the Likert scale feedback data received in the Qualtrics surveys; we report these findings as percentages (see Figure 4). Finally, we analysed the open-ended qualitative questions in two phases. We started by reading participant responses in real time during data collection to identify any concerning content/monitor the model’s responses to ensure participant safety and used this information to build a general understanding of participants’ experiences. Once the whole dataset was collected, we exported all qualitative survey feedback and conducted sentiment analysis[75] at the phrase level, where we manually analysed individual phrases of participant feedback for positive, negative, or neutral sentiment.

#### 4.1.2 Pilot #1 Results.

Participants’ overall reception of the interaction was positive across both the Likert scale question outcomes (see Fig 4) and the open text responses. For example, 90% reported the interaction was ‘very’ or ‘quite helpful’ in articulating their experience. Participants’ qualitative responses also highlighted the chatbot’s perceived ability to articulate personal experiences (e.g., *‘The chatbot was able to put all of my different thoughts together into one scenario and I was actually quite impressed with the results.’*) Similarly, when asked to qualitatively describe their experience of the interaction, the majority of participants (89%) responded positively (e.g., “very pleasant experience” “efficient”, “helpful” “useful” “easy and intuitive”) while some (6%) described the experience as neutral (e.g., “it was okay”, “chatbot standard”, “one-dimensional”). We identified five (5%) responses that contained explicitly negative language about the experience. Two of these were related to the pace of the interaction (“quite slow”, “tedious”), while two related to its emotional impact: “slightly aggravating”, “not at all like talking to a friend”. Finally, one participant mentioned the artificial nature of the interaction, amidst broader concerns about the use of GenAI, “talking to a chatbot about these experiences made me feel alone and unheard”. Although in the minority, these responses were of particular interest as they represented opportunities to improve the prototype.

##### Model Performance.

Overall, the scenarios accurately reflected participants’ answers to the questions and we identified no instances in which the model hallucinated (i.e., the resulting scenario would be different from the answers or experiences provided by participants). In our analysis of the output files, we identified some cases where the model added additional emotional details to the scenario not provided by participants – these were mostly situations where participants’ answers were very brief. For example, the response ‘it made me feel sad and lazy’ was presented as ‘it made me feel a bit sad and lazy like I wasn’t living up to my potential’ in the output scenario. Some participants provided feedback on this tendency, with mixed perspectives. Some participants (N > 10) saw it as a positive feature that helped capture their experience *‘It encapsulated my feelings quite well ... it took my prompts and expanded on them quite effectively.’* However, two participants were critical, commenting that the chatbot *‘exaggerated the story it told in some cases’* and *‘should try to be more accurate as it was putting in information. . . which I did not write about.’*

##### Ethics and Safety Concerns Exploration.

We probed participants’ perceptions of potential ethical and safety concerns about the method in two ways. First, we explicitly asked participants how they would feel about sharing real experiences with the chatbot and what concerns this would raise. Over half of participants (54%) said they would have no concerns, especially if appropriate privacy cautions were in place (e.g., *‘As long as it only asks about what had happened, but not any personal details, I would be fine with sharing my real experience.’*). The primary concerns raised by the remaining participants related to data privacy (e.g., *‘potentially have concerns as I wouldn’t know what’s happening with my data’*) and sharing sensitive information with AI systems. However, participants generally expressed being happy to share experiences as long as the chatbot didn’t *‘ask about personal details’*. Beyond privacy concerns, a small number (<5%) of participants raised more general ethical concerns about the use of AI, including an over-reliance on technology (e.g. *‘I am cautious about using AI for real-life problems’*) or *‘feeling weird ‘*about speaking to an AI system about personal problems. We also asked participants about their preference of method (survey, interview, chatbot interaction), if they were to complete the same study in the future. Most participants (58% ) said they would prefer the chatbot method over other data collection approaches. Finally, we asked participants to provide open-text feedback on how to improve the interaction, or what features they would remove. None of the resulting feedback suggested explicit concerns about the method. Instead, the key themes of feedback focused on the chatbot tone of data collection to better mimic human interaction (e.g., the ‘repetitive’ or too ‘generic and formal’ nature of the chatbot’s responding) and of the resulting stories.

Together these insights led us to design activities in Pilot #2 to probe these areas further, which is what we turn our attention to now.

### 4.2 Pilot Study #2 – Two-week Asynchronous Remote Communities Study

For Pilot Study #2, we designed a 2-week Asynchronous Remote Communities (cf.,[43, 44]) engagement (hereafter ARC) as a way to triangulate the user feedback from Pilot #1. The aims were to (1) test and further finetune the existing interaction based on Pilot #1 results (e.g., improve the ‘tone’ of the resulting stories and chatbot engagement); as well as to (2) better understand whether and how the micro-narrative process empowers the articulation of certain experiences for young people (as indicated by the open-text answers in Pilot #1), whilst also exploring any risks and benefits that would be inherent in this process.

We recruited N=30 Prolific users to take part in 4 different activities over a 14-day-period: The first two activities focused on understanding users’ feedback and preferences for the ‘voice’ (i.e., personas) that the system used to compose their narratives, and allowed the users to try five different personas. Detailed feedback on these personas is provided in the supplemental materials. The third and fourth activities aimed to capture more in-depth qualitative insights about the user’s experiences with the prototype, any changes to the design they would require, and users’ perception of future potential applications. We followed the same safeguarding procedure as in Pilot #1. See also Figure 7 for more details and participant numbers per activity. We also include the Miro board templates for each activity in the supplemental materials.

#### 4.2.1 Data analysis.

We undertook two streams of data analysis, reflecting the two primary aims of the ARC. First to understand participants’ preferences about the chatbot persona/voice (aim 1) we analysed the data from ARC activities 1 and 2, which consisted of participants’ chatbot interactions, survey feedback, and Miro activity boards. All data were imported into a single Miro board for analysis. Participant contributions were analysed using thematic analysis [5]. We took an inductive approach to coding, where the first author coded all participant contributions to identify key ideas and design suggestions. Authors 1 and 3 then collaborated on mapping codes into core themes and developing definitions. Authors 1, 3 and 10 met regularly throughout the analysis process to discuss the coding scheme and key themes. Second, we analysed the data from Activities 3 and 4, which were designed to examine if and how the micro-narrative process empowers young people’s articulation of their experiences (aim 2). These data consisted of interviews and Miro board responses. For interviews, data was audio recorded with permission from participants, and the recordings were stored in the University’s protected server. All interview data were anonymised before being transcribed and coded using thematic analysis (as described above). For Miro board data, data was analysed using the same thematic approach as for Activities 1 and 2.

#### 4.2.2 ARC Results.

Overall, the ARC findings supported and further expanded on the initial insights gained from Pilot #1. Across the activities, participants’ feedback echoed the generally positive sentiment we observed in Pilot #1, while also pointing to further design changes that could make it more engaging and youth-friendly. Additionally, the remote collaborative activities and interviews with youth helped us to gain a much richer understanding of how young people perceived the micro-narrative process as enabling articulation of their own stories; and the impact participants shared about it having on their own experiences. In the rest of this section, we discuss the more specific themes that emerged from the analysis of participant data across the ARC activities.

##### Participant preferences for narrative tone & language.

**Figure 6:**
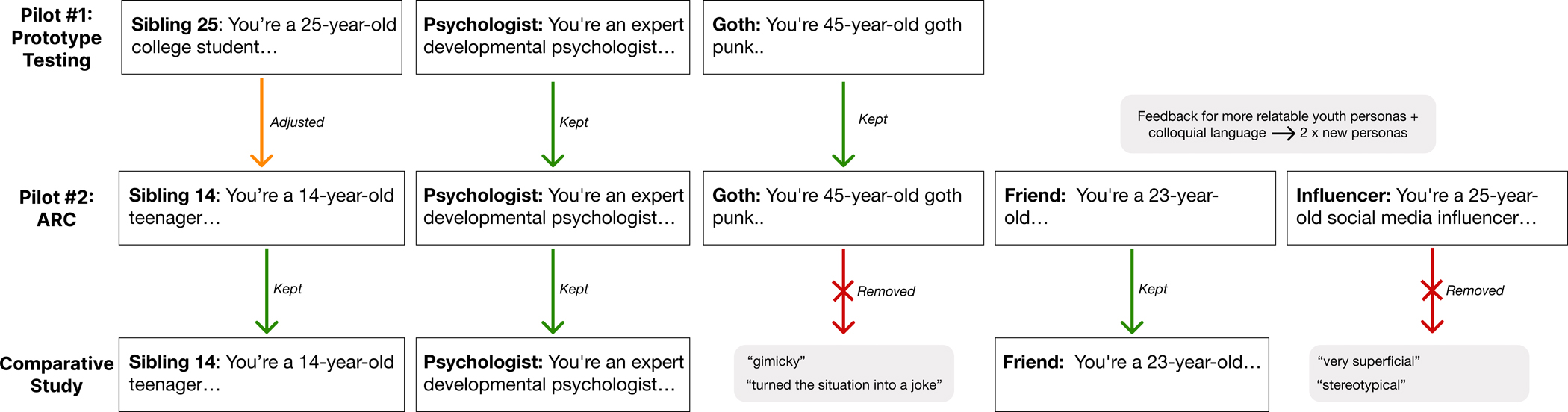
Summary of persona changes across the pilot and comparative studies

**Figure 7:**
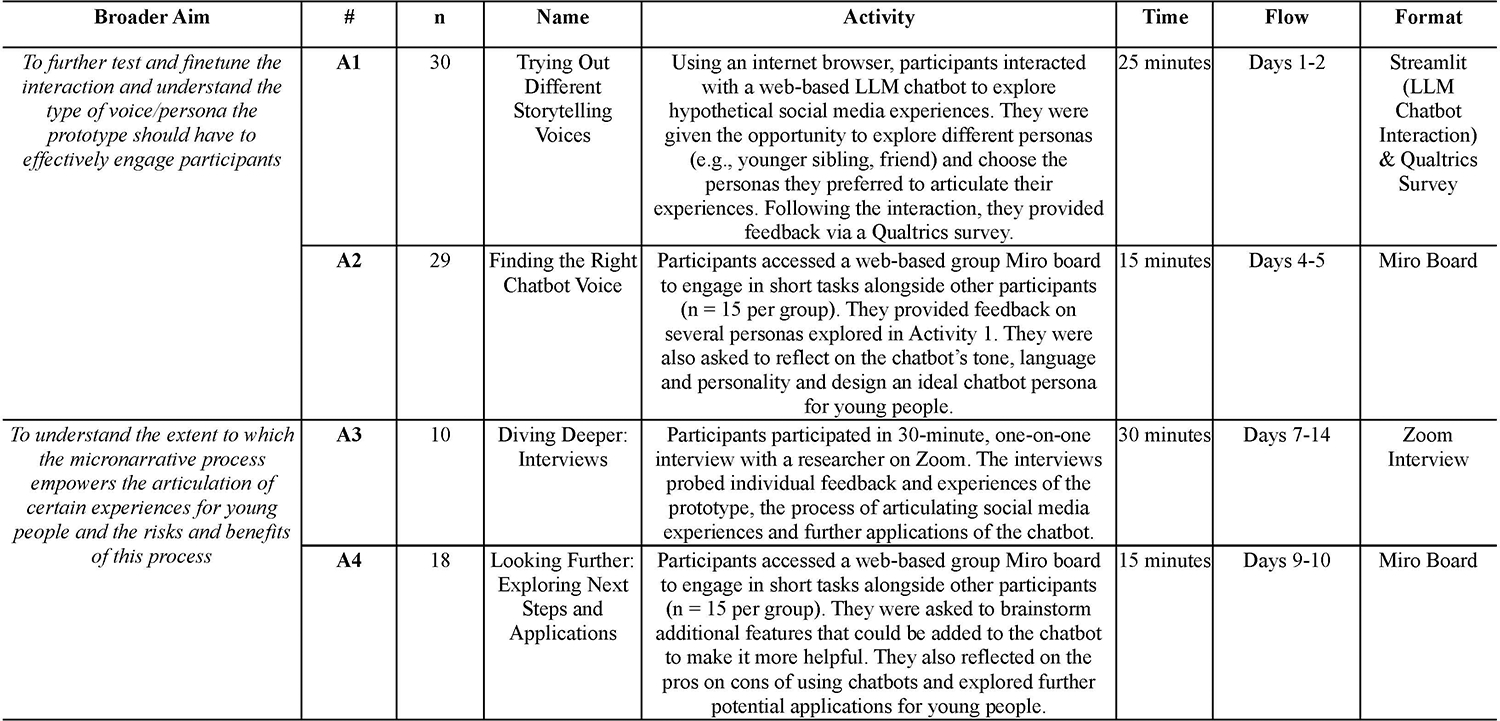
Summary of ARC activities

The use of appropriate slang and colloquial language was seen as important to effectively emulate young people’s voices. Participants noted that the personas distinguished the chatbot from other chatbots they had used (e.g., ChatGPT) which felt more rigid and impersonal – for example, one participant shared *‘It didn’t feel like a computer, it matches some people’s personality. The responses were like a certain type of person.’* Of the five sample personas that our participants could directly test and adapt (see Figure 6), the ‘friend’ was the most popular, likely because their age and tone were closest to those of the participants. This persona was described as relatable and realistic and this was deemed as important for ensuring the chatbot accurately captured participants’ tone in the interaction. For example, one participant described how the relatability of the persona influenced their perception of the chatbot’s ability to articulate their experience, *‘I related to this style much more, and felt as though it was my own thoughts being put on paper.’* In contrast, the goth persona was the most polarised and described as “gimmicky” and “unrealistic”. Together, these initial ARC engagements helped us to re-design the final set of personas for the main study, as well as gain further feedback on the importance of choice and relatability of the resulting narratives.

##### Understanding the micro-narrative articulation process and its perceived risks and benefits.

Participants often referred to the perceived value of the chatbot’s systematic, step-by-step questioning process, which helped them break down their experiences into manageable parts. One participant likened this to an exam process, where *‘you go through this part first, explore one feeling, and then move onto the next’* (P2). This structured approach not only seemed to make the process of reflection easier but also helped participants to organise or *‘streamline’* their thoughts more effectively. Several participants also stated that this led them to reflect more deeply on the experience and its emotional significance, *‘It was like, Oh, how did you feel about that? And I was like, oh, how did I feel about that?’* (P3). In such cases, participants described the resulting effect of engaging with their experiences as micro-narratives as one that could lead to *‘emotional realisations’* or *‘epiphanies’*. One participant described this as an immediate realisation, *‘After reading the scenario I realised that that is what I was thinking but prior to reading it I hadn’t really realised it’* (P9). Another participant characterised this interaction as a *‘sense-making’* process, where the act of seeing their experience echoed back to them, leading them to make sense of what had happened. Some participants also perceived this process as empowering, as it allowed them to voice their experiences in an *‘innovative’* way and helped them to *‘find the right words’* to articulate themselves. Two participants explicitly described the interaction as *‘therapeutic’* or *‘similar to a therapist’* as it allowed them to better understand their feelings and experiences. While participants saw this interaction as valuable for articulating *‘base and medium’* problems, they expressed some concerns about the chatbot being able to articulate deep personal issues, which may require more nuance and understanding of personal context. Indeed, when asked to reflect on the chatbot’s limitations, participants highlighted a lack of emotional depth and an absence of trust relative to human interactions (*‘can’t see what’s behind the screen compared to like a person you trust.’* (P1)). Finally, although we did not observe this in either of the two pilot studies, the potential for the chatbot to respond inappropriately or offensively was raised as a risk by participants, particularly if the tool was to be rolled out as part of a larger data collection process.

##### Youths’ perception of broader applications.

In Activity 4, we asked participants to reflect on other situations where the chatbot’s perceived articulation process may be helpful or unhelpful. Participants identified applications across a range of areas including navigating complex social dynamics, managing mental health, and addressing challenges related to identity, puberty and work. For example, participants suggested that the chatbot could assist in managing feelings of social exclusion. One participant mentioned the interaction’s utility for everyday conversations when they *‘struggle to find the correct words to convey what it is that I’m feeling and my emotional state means that I can’t find the words to articulate myself’* (P9). Reflecting on this ‘in situ’ approach, another participant expressed an interest in an app version of the prototype that could be easily accessed each time they needed to sound out a problem within their day. Younger adolescents were frequently identified as a group that would particularly benefit from these types of interactions as they might struggle more with articulating their feelings and emotions. Interestingly, participants’ applications centred largely around the interaction as a ‘sense-making’ tool, which—they thought—could be used to help them process or handle day-to-day emotional experiences.

### 4.3 Researcher reflections on pilot findings & summary of changes

Across Pilot Study #1 and Pilot Study #2: ARC, the findings were generally encouraging. As outlined in the previous subsections, participants predominantly responded positively to the presented prototypes. We also did not see any alarming instances of genAI malfunction across the 130+ micro-narrative examples generated by our participants. Participants also often described the human-AI interaction flow as being supportive of articulating their experience, and some even reported that the interaction enabled a perception of ‘sense-making’ or deeper reflection on their experience. These unintended impacts were reported by participants as positive side-effects in the pilot datasets – however, we were sensitive to the fact that there might be situations where similar insights could be detrimental (e.g., realising something negative and unexpected about oneself). In line with the positive reactions, the participants have asked for only limited changes to the prototype within the ARC process – as described above, our focus has been mostly on adapting the tone of the data-collection chatbot component (with minor adaptations to the prompt to lead to less formal dialogue) and the changes to the ‘persona voices’ available in the narrative building stage. These changes are illustrated in Figure 6.

Overall, we were satisfied that the adapted system could be taken into the validation stage, and that we could attempt to collect actual—rather than hypothetical—scenarios. To do so, we have further adapted and updated the safeguarding protocol, based on the insights above (especially around the double-edged sword of potential novel ‘sense-making’ effects) and additional safety risks of asking participants about real and potentially distressing experiences – see Section 5.1 for details.

## 5 Step 3: Comparative mixed methods study

**Figure 8:**
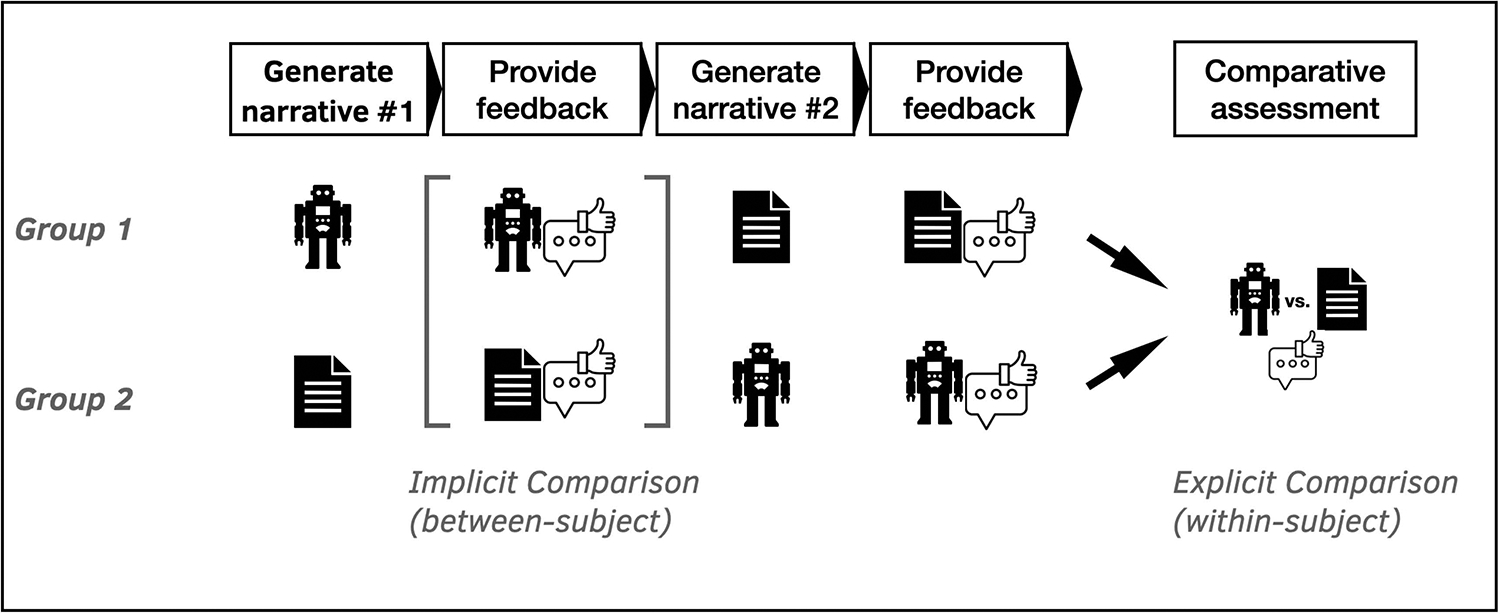
Overview of comparative study

Based on the positive findings from Pilot #1 and Pilot #2, we wanted to explore if the qualitative findings that indicated user acceptance and perceived support for articulation would hold even when participants are asked to *share their actual experiences*, rather than focusing on hypothetical examples. We were also particularly interested in investigating whether participants would see the articulation process as more or less simple/empowering/personally helpful when compared to the traditional open-text questionnaire question. We designed a comparative mixed methods study (hereafter comparative study) to examine how the micro-narrative elicitation flow—and the articulation interaction process it enables—compared to an open-form survey question (as the closest existing comparator^5^) – see Figure 8 for an overview. In what follows, we focus on the user *experience* of engaging with these two methods (i.e., micro-narrative elicitation vs open-text survey), and will not report on the *content* of the stories the users produced.

### 5.1 Method

#### Participants.

We recruited 269 UK-based participants through Prolific. We removed 15 participants who failed at least one of two attention checks (e.g., “Place the slider between 90–100”), leaving us with a final of 254 participants. We screened for participants between the ages of 18 and 20 and collected 64 participants of age eighteen, 86 participants of age nineteen, and 104 participants of age twenty (*M* = 19.16, *SD* = 0.80). We had a roughly even split of males and females (*n*_male_ = 124, *n*_female_ = 122, *n*_other_ = 8). Our sample consisted of 133 White individuals (52%), 77 Asian individuals (30%), 36 Black individuals (14%), 18 other (7%). Participants were allowed to select multiple racial/ethnic backgrounds.

#### Procedure.

**Figure 9:**
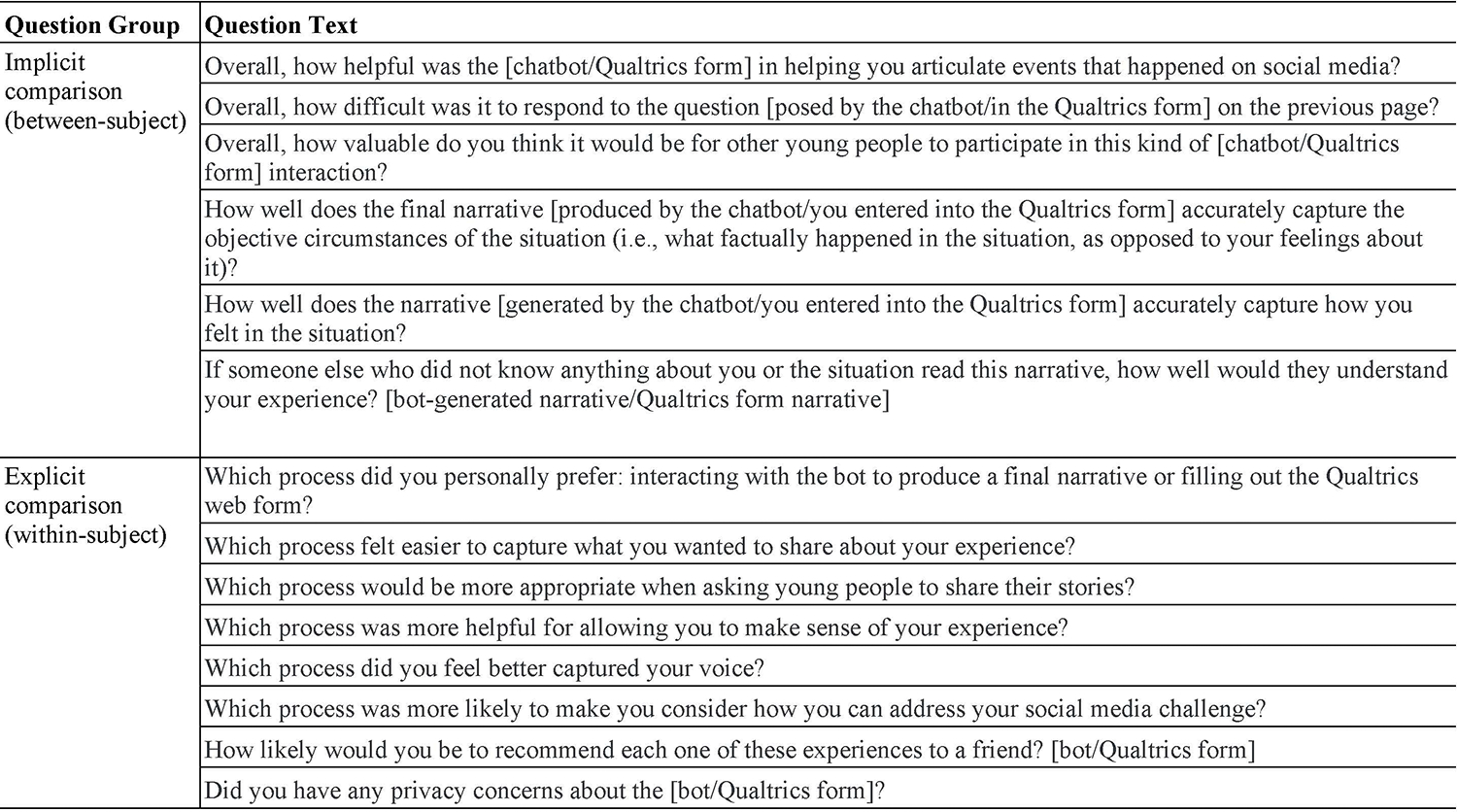
Wording of the survey questions as seen by participants. Question text in square brackets indicates the text that was different in different presentations of the same question. All questions in the implicit question group were answered on a unipolar scale (i.e., a single question refers to a single input method). In the explicit comparison group, “would recommend” and “privacy concerns” were reported on unipolar scales with separate questions for each input method. The rest of the questions in the explicit comparison group were reported on a bipolar scale where one side represented that the chatbot was higher on the given dimension and the other side represented that the Qualtrics form was higher on the given dimension.

Participants completed a three-part online study. In the first part, participants were randomly assigned to either fill out an open-text question or engage with the narrative elicitation flow described in previous sections (labelled as the ‘chatbot’ condition in the rest of this section). Next, they answered questions about their experience with their initially assigned condition. This allowed us to conduct between-subject analyses of the differences in people’s experiences with each of the two input methods without having been biased by the prior experience of the other condition. We refer to this set of analyses as the “implicit comparison”. In the second part, participants were assigned to do whichever activity they had not just completed (i.e., either form or chatbot) and then answered the same questions regarding their experiences with the second condition. Finally, in the third part, participants answered a series of questions asking them to explicitly compare the two experiences on several dimensions. We will therefore refer to these dependent variables as the “explicit comparison”. The wording of all questions is shown in Figure 9.

#### Safeguarding protocol.

Following the insights from pilot studies, we took several safeguarding steps in addition to the usual ethical requirements for these types of data collection studies (i.e., informed consent, voluntary withdrawal, optional responding). First, to mitigate data privacy concerns raised in the pilot studies, we instructed participants that they should not share any personally identifiable information during the chatbot interaction, including other people’s identifiable information (in addition to describing how data would be stored in the informed consent sheets). Second, all participant responses were monitored by researchers throughout the data collection stage with an established safeguarding plan in place for responses that showed evidence of suicidality/self-harm. (We note that no such ‘at risk’ responses were identified during this study). Third, we established clear communication pathways by providing researcher contact details at the beginning and end of the study (alongside an open-ended feedback box) and advised participants to contact us via Prolific for any time-sensitive issues. Fourth, we had mental health resources ready to provide to participants if concerns were identified.

### 5.2 Data Analysis

#### 5.2.1 Implicit Comparison.

To analyse the responses to the implicit comparison segment of the study, we used Bayesian beta regression models estimated in the R package *brms* [6]. In our beta regression models, we regressed the two parameters of the beta distribution, the mean parameter *μ* and the precision parameter *ϕ*, onto a binary variable indicating whether the data point corresponded to the Qualtrics form condition (coded as 0) or the chatbot condition (coded as 1). For the purposes of the present study, we were primarily interested in the effect of condition on *μ*, the mean of the beta distribution. Thus, the parameter that we report indicates the difference in means between the chatbot condition and the Qualtrics form condition, such that positive values indicate that the chatbot had a greater mean and negative values indicate that the chatbot had a lower mean.

#### 5.2.2 Explicit Comparison.

In the explicit comparison portion of the study, we used two styles of response scales – unipolar scales where participants completed a separate question for each of the two input methods (chatbot and Qualtrics form) and bipolar scales where one side referred to the bot and one side referred to the Qualtrics form (with counterbalanced assignment of conditions to sides of the scale). To analyse responses on unipolar scales, we used beta regression models as described in our explanation of the data analysis for the implicit comparison questions. To analyse responses on the bipolar scales, we binarized responses for ease of interpretation and fit logistic regression models in *brms* [6]. Our logistic regression models were intercept-only models. Thus, the parameter of interest represents the log-odds that someone reported that the bot was higher on the given dimension than the Qualtrics form. We exponentiated the log-odds in our results for ease of interpretation. We used *brms* default, weakly informative priors for all models presented in the manuscript.

### 5.3 Results

#### 5.3.1 Implicit Comparison Qestions.

We fit beta regression models to examine between-person differences between our chatbot and Qualtrics form for eliciting vignettes of negative experiences on social media. We found, generally, that the chatbot had more desirable characteristics than the Qualtrics form. Specifically, participants found the chatbot more helpful for articulating the experience (*μ*_difference_ = 22.04, 95% Cr.I. [17.70, 26.27]), less difficult to respond to (*μ*_difference_ = −5.58, 95% Cr.I. [−9.99, −1.17]), more valuable for youth (*μ*_difference_ = 5.40, 95% Cr.I. [0.15, 10.60]), more accurate for capturing the objective features of the situation (*μ*_difference_ = 11.75, 95% Cr.I. [7.43, 16.03]), more accurate for capturing their feelings (*μ*_difference_ = 14.51, 95% Cr.I. [9.91, 19.07]), and that others who read the final product would understand their experience better (*μ*_difference_ = 17.60, 95% Cr.I. [13.55, 21.63]).

#### 5.3.2 Explicit Comparison Qestions.

We fit beta regression models for two explicit comparison questions regarding privacy concerns and the probability of recommending each method to friends and found that people had more privacy concerns about the bot (*μ*_difference_ = 21.51, 95% Cr.I. [16.53, 26.56]), but were still more likely to recommend the bot to friends (*μ*_difference_ = 23.68, 95% Cr.I. [17.95, 29.25]). We fit intercept-only logistic regression models for the remaining five explicit comparison questions. Participants reported that the chatbot was easier to capture their experience (OR = 4.57, 95% Cr.I. [3.29, 6.28]), more appropriate for youth (OR = 2.86, 95% Cr.I. [2.14, 3.77]), better help in making sense of the experience (OR = 6.08, 95% Cr.I. [4.12, 8.54]), better captured their voice (OR = 1.79, 95% Cr.I. [1.35, 2.30]), and better elicited them to think about how to address their social media challenge (OR = 4.80, 95% Cr.I. [3.35, 6.71]).

#### 5.3.3 Findings summary and comparison to qualitative findings.

To summarise, participants preferred the chatbot tool for generating micro-narratives around their social media challenges. The chatbot was perceived as better at articulating their experiences and more accurately capturing both the objective and emotive aspects of these experiences. It was also deemed more appropriate for young people and seen to better capture participants’ voices and enable sense-making of their experiences, compared to the Qualtrics form. Despite having more privacy concerns about the chatbot than the form, participants were also more likely to recommend this data collection method. Overall, these findings support the qualitative insights we drew from Pilot #1 and Pilot #2 in relation to the key design assumptions. That is, participants positively experienced the LLM-supported articulation process and preferred this process over traditional, non-AI interactions. Additionally, this interaction was perceived as easier than building a narrative from scratch, better-enabling articulation in the user’s voice and more helpful for sense-making and personal insights when compared to traditional data collection approaches.

## 6 Step 4: Informal engagements with academic and non-profit experts

By engaging with a range of established researchers and non-profit leaders across a variety of fields, our aims were two-fold: First, to informally gauge the level of interest in the idea of collecting micro-narrative datasets (assuming a reliable tool was available) and the risks they perceived in doing so; and second, to gain an initial understanding of the types of research questions/design problems—if any—that this micro-narrative collection process might be uniquely well positioned to address. Given this approach, the rest of this section is not to be read as a traditional interview study (which would require a paper of its own), but rather as a set of informal engagements that served to help us, as designers, to start mapping out the potential use-cases and associated risks, which could be explored in future work. We note that these engagements took place *in parallel* to the studies described in Sections 4–5 – i.e., the expert’s reactions reported below included their own experiences with the prototype, prior to us having the positive data from the youth case study to report.

### Procedure.

We spoke with 14 established researchers (median citations 13.1k; range 1850–79500) across a range of international institutions^6^, and a spectrum of research domains^7^ that could benefit from such a tool. We also engaged with CEO/VP level staff from four non-profits across the UK, US, and Mexico, each serving more than 20k users, and several counting their reach in millions of young people.

Each expert was provided with a short email explanation and the opportunity to try out the current prototype over email, followed by an at least 30-minute-long Zoom call (or in-person conversation). Surprisingly, seven of the researchers and all of the four non-profits were immediately interested in exploring how the tool could be embedded into their ongoing research / data collection following the call. In these cases, the initial conversations turned into a thread of follow-up calls and emails (which are still ongoing at the point of submission). During the initial call, we were interested in understanding the experts’ immediate reactions to their interactions with the bot, how they imagined the collected data would be similar or different to interviews / surveys (or other methodologies they use on a regular basis), as well as what were their perceived benefits *and* risks if this approach was used in their contexts.

#### Observations and insights.

Overall, the experts seemed to value the ability to collect personal stories at scale as well as—often—showing a sense of surprise in how well the AI was able to combine their own words into fluent narratives. These insights then nearly always naturally progressed to reflections on how such data could be useful in their work. In what follows, we structure around four broad ‘types’ of use cases that consistently emerged across our various expert conversations.

1. Identifying **‘canonical’ stories**: The first type aligned with our original challenge of capturing personal narratives that address a specific, well-defined question from a large population. For example, some of our experts mentioned needing to understand the most salient challenge with accessing help (or lack thereof) for young people experiencing self-harm; or capturing examples of emergent good practice around large-scale behaviour change intervention implementation. In such examples, the micro-narratives were to be used to help distil the most commonly shared experiences, which would then be taken forward to guide further research (e.g., intervention development).
2. Gathering **multi-story collections**: Some of the experts were interested in the opportunity to collect multiple stories from each participant over a longer period (e.g., daily or weekly), as a way to create a more holistic understanding of participant experiences. For example, one of our experts was interested in collecting a series of micro-narratives to understand more about what it feels like to live with obesity for a specific marginalised US population – including stories about ‘a time they felt like giving up’, ‘a time when someone said or did something that really helped on their journey’, or a ‘time when they felt pressured to lose more weight then felt healthy for them’. We note that, ideally, at least some such target story ‘stems’ within the collection would be co-produced by members of the community to capture the type of narratives that the participants themselves want to share.
3. Capturing **intervention outcomes**: Some experts perceived micro-narratives as an innovative approach to study the effects of interventions at scale (e.g., process analysis capturing most people within an intervention), while remaining highly personalised and open-ended to capture patients’ needs; or even driving intervention adaptation (e.g., as an input into JITAI decision algorithms).
4. Micro-narrative as **part of interventions**: Finally, some of our experts were interested in collecting individual stories directly as part of the active intervention components, rather than ‘just’ as an empirical data collection. They envisioned such micro-narratives as a novel approach to potentially amplify existing intervention approaches (e.g., as an input into a weekly patient-therapist conversation, or driving a goal-setting process in behavioural change), or to perhaps serve as an intervention of its own (e.g., as part of enabling reflective practice or as a cognitive distancing intervention).

The excitement about the *potential* of similar micro-narrative techniques was understandably tempered by safeguarding and data protection questions, as well as concerns about potential hesitations from respective IRB / ethics committees – at least until this LLM-driven method is ‘standardised’. Our perception was that the experts saw these issues as crucial steps that would require deep and thoughtful work as these methods will continue to be developed, but did not see any insurmountable risks. We discuss the expert perspectives and our own reflections from the empirical studies on risk and ethical questions in more detail in the Future Research Agenda subsection of the discussion (Section 7).

## 7 Discussion

The purpose of this paper has been to try to develop a novel data collection technique— micro-narrative elicitation—that would provide a new way of approaching the trade-off between the richness of the collected datasets, and the required effort from researchers / participants to do so. In what follows, we first briefly revisit our empirical findings and then move on to discussing the (many!) remaining open questions that will need to be resolved.

### Summary of results across studies.

Our design explored the potential of a novel human-AI flow to scaffold users’ articulation of short narratives about their lived experience. Three key design assumptions drove our development and design work: First, the *users’ articulation process* can be based on a template-based vignette structure – enabling the researchers to specify the structure of the story, but allowing the participants to fill it in with their own words (DA1). Second, the *cognitively burdensome parts of this process could be supported through LLM-powered components*, while fully *retaining the participants’ words and control* over the resulting narrative (DA2). And finally, *such a process would be ideally perceived as simple, empowering, and insightful* by the participants (DA3).

Across our pilots (Pilot #1 (N=100), Pilot #2 (N=30)), and the comparative study (N=254) the findings demonstrated the acceptability & potential for micro-narrative elicitation as a new approach to collecting narrative data about participants’ lived experiences. Overall, the participants’ feedback described the approach as a potentially ‘helpful’ way for participants to articulate a personal experience in a way that ‘makes sense’. Some participants also described the process as empowering and personally meaningful, and the comparative study showed micro-narratives to be perceived better than an equivalent open-text survey interaction. Further research is however needed to understand how consistently these perceptions hold across diverse groups, types of stories collected, and how they may interact with participants’ comfort or trust in the system.

Moreover, our informal engagements with experts in non-profit and academic domains suggested the potential that such narrative elicitation interactions (as data collection processes) could have across multiple research domains, and the range of varied use cases that the experts were interested in exploring was unexpected. We note that our initial attempts to adapt the tool to elicit narratives in other domains (e.g., diabetes) were surprisingly simple – only requiring the change of the list of questions in the Data Collection component, and the narrative template in the Narrative Creation LLM. A refactored codebase now allows such changes to be specified as a textual ‘config’ file, enabling researchers to quickly iterate and test the resulting system. However, the actual value and applicability of micro-narratives in other areas remain to be tested and warrant careful investigation to determine their feasibility, ethical considerations, and the safeguarding components required for practical use.

Although the experience was designed (and presented to participants) as a data-collection approach, the process of articulating a narrative with the system appeared to help some participants to make sense of or emotionally reflect upon the experience. This indicates the potential for the method to generate additional benefits—and risks—over and above the contribution of participants’ personal data. Such suggestions came directly from participants’ data (e.g., personal ‘sense-making’ benefits described above as well as the broader implications imagined in the last ARC task); as well as some of our expert conversations (e.g., envisioning micro-narratives as part of mental health interventions). We see these opportunities as an exciting potential direction for future work – but also want to emphasise that there is, so far, very little known about the potential risks (and benefits) of adapting similar human-AI collection flow specifically for mental health interventions. We argue that any such research will require the involvement of clinical psychologists and strict adherence to guidelines of digital mental health intervention development (cf., [12, 39, 50, 51, 82]), as well as engagement with existing theory in clinical science around the potential psychological impacts of narrative creation in mental health (cf., [23, 52, 57, 85]). Importantly, the potential for such (unintended) mental health side-effects raises the bar on the need for considerate and rigorous safeguarding approaches, even if micro-narratives are intended to be deployed as a data collection technique only, and especially in sensitive or emotionally difficult contexts.

### Relationship to other data collection methods.

We want to be clear that we *do not* expect the micro-narrative elicitation method proposed here to replace any existing approach to data gathering, whether in HCI or more broadly; or attempt to make comparisons in terms of their value for design in general. Instead, this approach should be seen just as an *additional tool*, one that is complementary to well-known methods, and that might—at times—be well-suited to particular design questions. In our view, any potential benefits of this method stem simply from a different approach to balancing data richness vs. effort: on one hand, the proposed process is highly constrained by its ability to *only* capture a highly pre-specified aspect of the user’s experiences (that fit the proposed vignette template); on the other, it is exactly this narrow focus that then allows us to provide streamlined support to simplify users’ articulation of their stories. Such a scaffolded approach to narrative articulation then raises several open questions about the ethics and epistemological considerations around the nature of the data collected, which is what we turn to in the next section.

#### Open questions and research agenda

##### Safeguarding and ethical use – as data collection:

Any data collection method that aims to capture participants’ experiences around sensitive issues must carefully consider key questions around data safeguarding and other ethical risks – and many well-researched approaches to safeguarding protocols are already available within the psychological literature (e.g., in domains such as self-harm or suicidal ideation, cf., [42]). Similarly, substantive literature has already engaged with the questions of the use of AI in data collection (cf., [62, 76]) as well as other highly sensitive contexts (such as AI use in mental health therapy [68]). Based on our discussions with experts and our own reflection, we would like to highlight four main directions that any use of this—or similar methods—should consider.

First, when probing sensitive personal experiences, particularly those surrounding mental well-being, *there is a risk of participants* *disclosing sensitive information*. The magnitude of such risk clearly differs across topics (e.g., asking for teachers’ best practices is less risky than collecting stories of mental health help-seeking). However, such risk will increase with larger sample sizes and is particularly amplified for vulnerable populations, such as youth and adolescents (cf., for example [63]). Researchers should therefore implement appropriate safeguarding protocols alongside the deployment of these tools to mitigate disclosure risks and ensure researchers respond appropriately and efficiently to disclosures^8^. The best practices available will differ depending on the researched topic and domain: In high-risk settings, this will likely require including a trained therapist ‘on-call’ to review the data as it is being collected (cf., [33]), which is common in clinical settings including large-scale out-patient trials [56]. In lower-risk settings, further research could explore the possibility of including additional safeguarding modules, implemented within the Data Collection LLM component that would check the dialogue for risks, and invoke safeguarding procedures—such as diverting to another interaction flow, providing help resources, or alerting a human therapist—as necessary.

A second open question concerns LLM responses and the potential for LLMs to *respond insensitively or detrimentally* *to participants’ experiences*. While our approach inherently mitigates this risk by strongly scoping the interaction (to only include specific, pre-determined questions), there is again a need to consider additional safeguarding, particularly for vulnerable and marginalised populations. This is an active area of research [38, 83], with a wide range of techniques available depending on the research contexts and specific risks.

Third, the *ethics of* *developing a ‘listener’ AI*—i.e., an interaction that is seen as supportive and potentially validating—have not been fully explored in research and further exploration is needed to understand how these tools may engage and affect the participants who use them – and in the context of this work, also shape the narratives being produced (cf., [25]). This is particularly important in light of the present findings, where some youth described the interaction as therapeutic/supportive, despite this not being a design aim. We see this as a crucial area for future research, and one which can draw on existing work around therapeutic chatbots – cf., [18] for an extensive recent review.

Finally, we highlight the issue of *domain-specific ethical risks* that are present in LLM interactions broadly but might become particularly pronounced as part of the narrative articulation process here. For example, while LLM development often involves deliberate fine-tuning of the model to affirm the user’s perspective, such interactions could be harmful in some mental health contexts (cf., [39]). Consider a participant with depression stating, “I have no friends and everyone hates me.” If the system reflects this back as part of the story, it might inadvertently validate participants’ negative self-perceptions as factual truths rather than subjective beliefs. These concerns will require researchers to carefully balance the knowledge of their research context and target users with the ability to integrate mental health guidelines into the system (or safeguarding) design so as not to inadvertently support participants in reifying harmful distortions.

##### Epistemological questions:

The conversations with experts opened a range of questions about how one could understand, interpret, and analyse the micro-narrative data. On one hand, more research is needed to understand the extent to which the data—co-created as a part of a human-AI workflow—is indeed capturing what the participant has *meant to* articulate; and how we should interpret such data in situations where they would not have been able to describe such a story without such help. Further, there are epistemological challenges of working out how one might analyse/interpret large datasets (e.g., 100s of micro-narratives) and how this relates—if at all—to the more traditional iterative and discursive approaches used in HCI (cf., Section 2.1). Finally, the resulting narrative is only part of the data that could be captured. It is possible for the system to also collect the highly structured ‘provenance’ that led to the narrative—including, for example, the verbatim text written by the participants, the extracted content LLM used, the choices made by the participants, and any follow-up adaptation requests / edits—all of which could be used for computational analysis, for example, similar to what is currently done on large-scale Reddit datasets.

##### Broader design questions.

In principle, an abstracted description of the design work here is focused on a specific cognitive flow (i.e., articulating one’s experience into a narrative form that includes predetermined aspects of the situation), which has been seen as important but also difficult for many participants to do. The design itself then ‘only’ reframed such cognitive flow interaction into components that seemed ‘easy’ for the human to do (answer questions about fragments; make a choice between scenarios as to which one seems closest to own perception; ask for adaptation) while ensuring that the remaining components were ‘easy’ to do for the computational system (ask X questions in a row; extract information; combine information following a template). Some of the envisioned use-cases by the experts are already pointing to extensions that could rely on similar design patterns. For example, the suggestions to use the micro-narratives as a cognitive distancing intervention (cf., [15]) would likely include retaining the story generation (current process), but then extending it with components that could, e.g., address the challenging parts of the ‘decentering’ metacognitive process; as a core-but-difficult cognitive flow within the existing intervention approach.

We expect that there must be many other important cognitive flows that could be decomposed in analogous ways, and lead to innovative HCAI systems that are based on a deep understanding of the learning / cognitive challenges that users experience (cf., [67]) and the understanding of the strengths that (agentic) LLM systems could bring into the process.

## 8 Conclusion

This paper introduces a novel, gen-AI supported data collection method—micro-narratives—which could help collect rich but narrowly scoped qualitative data at scale. Specifically, the method aims to empower participants to easily articulate their stories in their own voice, regardless of their writing ability. To accomplish this, the method leverages AI chaining to enable the design of a scaffold that breaks the creation of such narratives into steps that are easy for individuals to accomplish, automates aspects of narrative creation that are difficult, while giving individuals agency to revise the resulting narratives to make them their own. Both qualitative and quantitative results showcase the acceptability and feasibility of this data collection approach within a particular case study; while expert engagements point to a wide range of potential additional applications. Many ethical and epistemological questions remain to be addressed in future work before this promising data-collection approach can be deployed at scale.

## Supplementary Material

Appendix A includes all the gen-AI prompts used in the proof-of-concept system.

Appendix B includes all study protocols and results for the two user-centred pilots. We also include more detailed persona feedback from the ARC study and all tables from the main manuscript text as editable tables

## Figures and Tables

**Figure 3:**
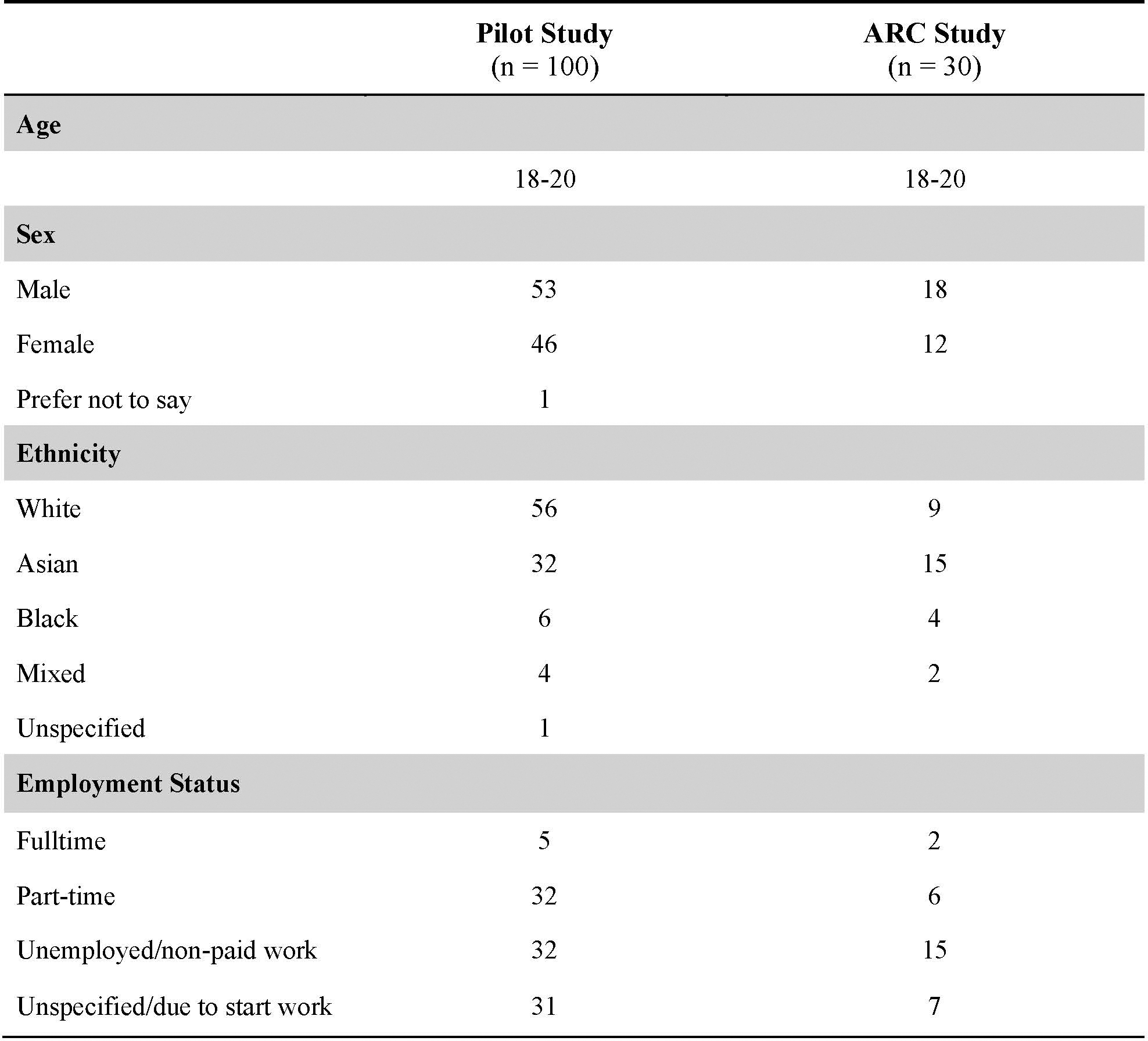
Demographics for user-centred pilot studies

## CCS Concepts

• Human-centered computing → User centered design.

## Footnotes

1 Note that by within-subject comparison, we refer to the fact that participants were making a within-subject judgement between within-subject conditions. However, since participants provided one response (on a bipolar scale), the model was still a between-subjects model.

2 We note that the flexibility and wide-spread interest in probes has meant that interaction design researchers have taken on and adapted probes in many ways, leading towards more varied applications as well as epistemological and conceptual disagreements on what the role / form of probes could be – see for example [4, 86]. For the purposes of our argument here, we will point the reader to the literature that differentiates among, for example cultural probes [20], technology probes [27], empathy probes [47] and informational probes [11].

3 Such as in the areas of understanding trustworthiness & transparency of AI decision making – see, for example, [2] for a well cited instance, examining the impact AI-decision explanation styles (input, sensitivity, case-based, demographics) have on perceived fairness of the resulting decision; or [9, 13, 73, 74] for other CHI examples from last two years.

4 As an example, the Institute for Sustainable Futures toolkit includes a similar activity flow, which is intended to be administered as part of the facilitator-led workshops – see link here.

5 We aimed to keep the open-text question as close as possible to the template used within the micro-narrative flow. Participants were informed that we were *collecting experiences about challenging situations that people have experienced on social media. Please think of a situation that you experienced on social media that you felt was challenging or troubling.’* They were then asked to *‘Please tell us about this experience and be sure to include details about: what happened, what was said, posted, or done, what was the context of the situation, if there is anything else that should we know about the situation, how the situation made you feel, how you reacted, and what the worst part of the situation was.’*

6 Cambridge, Harvard, King’s College London, LSE, University of Melbourne, University of Michigan, Monash University, Northwestern University, University of Nottingham, University of Oxford, University of British Columbia, University of California Irvine, University of Washington

7 The broad domains included psychology, clinical psychology (including researchers working on self-harm/suicide interventions), implementation science, health behaviour change, and HCI.

8 We note that research has demonstrated that, even in high-risk scenarios such as discussing suicidality, asking openly about these topics does not increase suicidal ideation [14]; and could in some cases even lead to reductions in suicidal thoughts and an increased willingness to seek treatment [3] – if provided within a supportive safeguarding framework. For example, trauma-informed care principles reinforce the importance of ensuring that participants maintain control over their engagement [36], choosing when and how to share their narratives, and having the option to stop at any point.
