# Supplementary material for: Micro-narratives: A Scalable Method for Eliciting Stories of People’s Lived Experience
Source: Proc SIGCHI Conf Hum Factor Comput Syst. Author manuscript; Available in PMC 2025 Jul 16. (PMC12265993; doi:10.1145/3706598.3713999)
Supplement: Appendix B includes all study protocols and results for the two user-centred pilots. We also include more detailed persona feedback from the ARC study and all tables from the main manuscript text as editable tables [file NIHMS2083225-supplement-Appendix_B_includes_all_study_protocols_and_results_for_the_two_user-centred_pilots__We_also_include_more_detailed_persona_feedback_from_the_ARC_study_and_all_tables_from_the_main_manuscript_text_as_editable_tables.docx]

**Supplemental 1 - Prototype Pilot Survey - Qualtrics**

Start of Block: Screener Validation

Age How old are you?

- 18 (3)
- 19 (4)
- 20 (5)
- Others, please specify: (6) __________________________________________________

Q17 Where do you currently reside?

- United Kingdom (1)
- Other, please indicate: (2) __________________________________________________

End of Block: Screener Validation

Start of Block: Demographics

Gender How would you describe your gender identity?

- Cisgender Male (1)
- Cisgender Female (2)
- Non-binary (3)
- Transgender Female (4)
- Transgender Male (5)
- Other: please specify (6) __________________________________________________
- Prefer not to say (7)

Household income What was your total household income before taxes for the last 12 months?

- Less than £20,000 (1)
- £20,000 - £39,999 (2)
- £40,000 - £59,999 (3)
- £60,000 - £79,999 (4)
- £80,000 - £100,000 (5)
- Over £100,000 (6)
- Prefer not to say (7)

Ethnic What is your ethnicity?

- White (1)
- Mixed/multiple ethnic groups (2)
- Asian/Asian British (3)
- Black/African/Caribbean/Black British (4)
- Other ethnic group: please specify (5) __________________________________________________
- Prefer not to say (6)

End of Block: Demographics

Start of Block: SM and LLM questions

Q7 Do you use social media?

- Yes (1)
- No (2)

| Page Break |  |
| --- | --- |

Q8 Which social media platforms do you use? You can select multiple platforms.

- TikTok (1)
- Instagram (2)
- Snapchat (3)
- Whatsapp (4)
- Facebook (5)
- Discord (6)
- Reddit (7)
- Youtube (8)
- Others: please specify (9) __________________________________________________

| Page Break |  |
| --- | --- |

Q9 What are the main reasons you use social media?

________________________________________________________________

| Page Break |  |
| --- | --- |

Q10 What are the main reasons for not using social media?

________________________________________________________________

| Page Break |  |
| --- | --- |

Q11 How often do you use social media?

- Daily (1)
- A few times a week (2)
- A few times a month (3)
- Rarely (4)

| Page Break |  |
| --- | --- |

Q12 Do you use ChatGPT or any other LLM tools (e.g., Microsoft Bing AI, Gemini)?

- Yes (1)
- No (2)

| Page Break |  |
| --- | --- |

Q13 Which LLM tools do you use? You may select multiple answers.

- ChatGPT (4)
- Claude (5)
- Google Bard (7)
- Google Gemini (8)
- Meta Llama 3 (12)
- Microsoft Bing AI (13)
- Perplexity (16)
- Snapchat My AI (17)
- Others, please specify: (18) __________________________________________________

| Page Break |  |
| --- | --- |

Q14 What are the main reasons you have used LLM tools?

________________________________________________________________

| Page Break |  |
| --- | --- |

Q15 What are the main reasons why you have not used LLM tools?

________________________________________________________________

Q16 How often do you use LLM tools?

- Daily (2)
- A few times a week (3)
- A few times a month (4)
- Rarely (5)

| Page Break |  |
| --- | --- |

Q17 Have you encountered any challenges when using LLM tools?

________________________________________________________________

End of Block: SM and LLM questions

Start of Block: Pre-Protoype Probing

Q18 The goal of this project is for researchers to collect data on young people's social media experiences in the form of stories or 'micro-narratives'. Below is an example of what these stories might look like: **Social Media Story Example** *I’ve been feeling guilty about social media lately. I've been staying up super late watching TikToks, even when I know I have to get up early for school or work the next day. It makes me feel like I’m addicted, sometimes I even wake up in the night and start to watch TikToks. The worst part is, it feels like I’m being controlled by social media. I can't stop watching these videos, even when I feel tired all the time.*  We want to work together with young people to collect these kind of social media stories and learn more about how social media is challenging for them.  To what extent does this research seem valuable to you.

|  | Not valuable at all | Slightly valuable | Moderately valuable | Very valuable | Extremely valuable |
| --- | --- | --- | --- | --- | --- |

|  | 1 | 2 | 3 | 4 | 5 |
| --- | --- | --- | --- | --- | --- |

| 1 () | 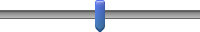 |
| --- | --- |

| Page Break |  |
| --- | --- |

Q19 **Social Media Story Example** *I’ve been feeling guilty about social media lately. I've been staying up super late watching TikToks, even when I know I have to get up early for school or work the next day. It makes me feel like I’m addicted, sometimes I even wake up in the night and start to watch TikToks. The worst part is, it feels like I’m being controlled by social media. I can't stop watching these videos, even when I feel tired all the time.*  Imagine someone asks you to write this type of social media story. The story should include information on **what happened, any context the reader needs to know, how it made it you feel** and **how you reacted.** How much effort would it take for you to write this story from scratch using these prompts?

|  | A lot of effort | No effort at all |
| --- | --- | --- |

|  | 1 | 2 | 3 | 4 | 5 |
| --- | --- | --- | --- | --- | --- |

| 6 () | 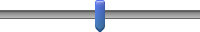 |
| --- | --- |

| Page Break |  |
| --- | --- |

Q20 **Social Media Story Example** *I’ve been feeling guilty about social media lately. I've been staying up super late watching TikToks, even when I know I have to get up early for school or work the next day. It makes me feel like I’m addicted, sometimes I even wake up in the night and start to watch TikToks. The worst part is, it feels like I’m being controlled by social media. I can't stop watching these videos, even when I feel tired all the time.*  If you had to create this story, how long do you think it would take you before you are happy with the story (and think it would be understandable to others)? Please provide your answer in minutes.

________________________________________________________________

| Page Break |  |
| --- | --- |

Q57 **Social Media Story Example** *I’ve been feeling guilty about social media lately. I've been staying up super late watching TikToks, even when I know I have to get up early for school or work the next day. It makes me feel like I’m addicted, sometimes I even wake up in the night and start to watch TikToks. The worst part is, it feels like I’m being controlled by social media. I can't stop watching these videos, even when I feel tired all the time.*  If you had to create this story from scratch, how annoying would you find this process?

|  | Very annoying | Pleasant |
| --- | --- | --- |

|  | 1 | 2 | 3 | 4 | 5 |
| --- | --- | --- | --- | --- | --- |

| 4 () | 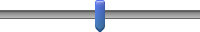 |
| --- | --- |

End of Block: Pre-Protoype Probing

Start of Block: Link to LLM

Q21 Great, **now please click on this link** (it opens in a new window) to access our LLM chatbot prototype. The chatbot will ask you about hypothetical challenges that young people face on social media. This process will take around 10 minutes. **Once the interaction is finished you will receive a code and be asked to return to the survey window. Please return and complete the rest of the survey.** We will ask your about your experience using the chatbot and get your feedback about how we can improve it.

End of Block: Link to LLM

Start of Block: PostSurvey

Q22 Thank you for interacting with our LLM prototype.  You received a code at the end of the final social media story, what was the code?

________________________________________________________________

| Page Break |  |
| --- | --- |

Q23 Overall, how helpful was the chatbot in helping you articulate events that happened on social media?

- Very helpful (1)
- Quite helpful (4)
- Neutral (5)
- Not helpful (6)
- Very unhelpful (7)

| Page Break |  |
| --- | --- |

Q24 Overall, how well did the chatbot to help you formulate your thoughts around specific social media challenges?

- Very well (1)
- Quite well (4)
- Neutral (5)
- Not well (6)
- Not well at all (7)

| Page Break |  |
| --- | --- |

Q26 Imagine a researcher asks you to create a social media story using the chatbot process you just trialled.   How much effort would it take for you to create a social media story using the chatbot?

|  | A lot of effort | No effort at all |
| --- | --- | --- |

|  | 1 | 2 | 3 | 4 | 5 |
| --- | --- | --- | --- | --- | --- |

| 1 () | 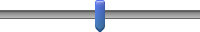 |
| --- | --- |

End of Block: PostSurvey

Start of Block: OpenEnded

Q27 If there was one thing you could improve about your general experience of interacting with the chatbot, what would it be?

________________________________________________________________

| Page Break |  |
| --- | --- |

Q28 Are there any specific features of the chatbot that you think should be improved or removed?

________________________________________________________________

| Page Break |  |
| --- | --- |

Q29 If you were to describe your experience of interacting with the chatbot to a friend, what would you say?

________________________________________________________________

| Page Break |  |
| --- | --- |

Q30 The chatbot prototype involved hypothetical stories. If the chatbot was to ask you about**real** experiences that you've had on social media, how would that make you feel? Would you have any concerns about this?

________________________________________________________________

| Page Break |  |
| --- | --- |

Q31 If you had to take part in this type of study again, would you prefer the story creation process to be:

- a survey asking about you questions about your social media experiences (1)
- an interview with a researcher who asks you questions about your social media experiences (2)
- a chatbot that interacts with you to ask you questions about your social media experiences (3)

End of Block: OpenEnded

Start of Block: endofsurvey

Thank you for your interest in our research project. You will shortly be redirected back to Prolific and receive more information about payment. If you have any questions about this study, please feel free to contact amira.skeggs@mrc-cbu.cam.ac.uk or valerie.yap@mrc-cbu.cam.ac.uk.

End of Block: endofsurvey

**Supplemental 2 - Results – Pilot Study**

Q57 **Social Media Story Example** *I’ve been feeling guilty about social media lately. I've been staying up super late watching TikToks, even when I know I have to get up early for school or work the next day. It makes me feel like I’m addicted, sometimes I even wake up in the night and start to watch TikToks. The worst part is, it feels like I’m being controlled by social media. I can't stop watching these videos, even when I feel tired all the time.*  If you had to create this story from scratch, how annoying would you find this process?

|  | Very annoying | Pleasant |
| --- | --- | --- |

|  | 1 | 2 | 3 | 4 | 5 |
| --- | --- | --- | --- | --- | --- |

| 4 () | 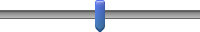 |
| --- | --- |

**Result:**

**Mean** = 2.82

Q23 Overall, how helpful was the chatbot in helping you articulate events that happened on social media?

- Very helpful (1)
- Quite helpful (4)
- Neutral (5)
- Not helpful (6)
- Very unhelpful (7)

| Page Break |  |
| --- | --- |

**Result:**

**Response Count**

1 Neutral 7

2 Not helpful 2

3 Quite helpful 41

4 Very helpful 49

5 Very unhelpful 1

Q24 Overall, how well did the chatbot to help you formulate your thoughts around specific social media challenges?

- Very well (1)
- Quite well (4)
- Neutral (5)
- Not well (6)
- Not well at all (7)

| Page Break |  |
| --- | --- |

**Result:**

**Response Count**

1 Neutral 8

2 Not well 1

3 Not well at all 1

4 Quite well 49

5 Very well 41

Q26 Imagine a researcher asks you to create a social media story using the chatbot process you just trialled.   How much effort would it take for you to create a social media story using the chatbot?

|  | A lot of effort | No effort at all |
| --- | --- | --- |

|  | 1 | 2 | 3 | 4 | 5 |
| --- | --- | --- | --- | --- | --- |

| 1 () | 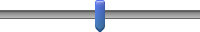 |
| --- | --- |

**Result:**

**Mean** = 3.69

Q31 If you had to take part in this type of study again, would you prefer the story creation process to be:

- a survey asking about you questions about your social media experiences (1)
- an interview with a researcher who asks you questions about your social media experiences (2)
- a chatbot that interacts with you to ask you questions about your social media experiences (3)

End of Block: OpenEnded

**Result:**

**Response** **Count**

1 chatbot 57

2 interview 8

3 survey 34

Missing = 1

Supplemental 3 - ARC – Activity 1 - PRE SURVEY (Qualtrics)

Start of Block: Screener Validation

Age How old are you?

- 18 (3)
- 19 (4)
- 20 (5)
- Others, please specify: (6) __________________________________________________

Q17 Where do you currently reside?

- United Kingdom (1)
- Other, please indicate: (2) __________________________________________________

End of Block: Screener Validation

Start of Block: Demographics

Household income What was your total household income before taxes for the last 12 months?

- Less than £20,000 (1)
- £20,000 - £39,999 (2)
- £40,000 - £59,999 (3)
- £60,000 - £79,999 (4)
- £80,000 - £100,000 (5)
- Over £100,000 (6)
- Prefer not to say (7)

Ethnic What is your ethnicity?

- White (1)
- Mixed/multiple ethnic groups (2)
- Asian/Asian British (3)
- Black/African/Caribbean/Black British (4)
- Other ethnic group: please specify (5) __________________________________________________
- Prefer not to say (6)

End of Block: Demographics

Start of Block: Link to LLM

Q21 Great, now please click on this link (it opens in a new window) to access our LLM chatbot prototype and start the study activities. The study has two parts:  **1. Social Media Story:** the chatbot will ask you about hypothetical challenges that young people face on social media. You will answer a series of questions from the perspective of a young social media user.  **2. Trying Out Different Voices:**for the second part of the task we want you to try out different personas for the chatbot. Please take your time to complete this part and try out several personas. You will be asked a series of follow up questions about this experience once it is completed.    Once you have completed both parts of the task, **please return to this survey window.** You should **keep the chatbot window open** while you complete the rest of the survey**.** We will ask your about your experience of creating new personas and get your feedback on the chatbot interaction. This process should take around 20 minutes.

End of Block: Link to LLM

Start of Block: OpenEnded

Q27 Thank you for completing the task trying out different personas for the chatbot. You should keep the chatbot window open to help you answer the following questions.  Could you please tell us which persona or voice was your favourite? This can be one that you created yourself or one of the samples provided (e.g., younger sibling).

________________________________________________________________

| Page Break |  |
| --- | --- |

Q28 What did you like about this persona? Why was it your favourite?

________________________________________________________________

| Page Break |  |
| --- | --- |

Q29 Could you please tell us which persona or voice was your **least**favourite? This can be one that you created yourself or one of the samples provided (e.g., younger sibling).

________________________________________________________________

| Page Break |  |
| --- | --- |

Q30 What did you **dislike** about this persona? Why was it your **least** favourite?

________________________________________________________________

| Page Break |  |
| --- | --- |

End of Block: OpenEnded

Start of Block: Block 8

Q58 **Imagine you were creating this chatbot to help young people create stories around their experiences of social media.**  What should the chatbot sound like to authentically reflect young people's experiences? Could you describe the ideal voice you would want this chatbot to have.

________________________________________________________________

| Page Break |  |
| --- | --- |

Q59 **Returning to the current chatbot personas (you can review your scenario history to help you).** How did the sample personas compare to the ones you made yourself? What elements did you focus on changing?

________________________________________________________________

| Page Break |  |
| --- | --- |

Q62 Of all the personas, which one do you think most authentically reflected young people's voice about social media experiences?  Please list one persona. This can be one that you created yourself or one of the samples provided (e.g., younger sibling).

________________________________________________________________

| Page Break |  |
| --- | --- |

Q60 Where did the current chatbot personas fall short? Is there anything you felt was missing from the voices you tried out?

________________________________________________________________

| Page Break |  |
| --- | --- |

Q61 Generally, is there anything that was unclear about the persona task? Or anything you felt like you wanted to change but didn't know how?

________________________________________________________________

End of Block: Block 8

Start of Block: endofsurvey

Thank you for your interest in our research project. You will shortly be redirected back to Prolific and receive more information about payment. If you have any questions about this study, please feel free to contact amira.skeggs@mrc-cbu.cam.ac.uk or valerie.yap@mrc-cbu.cam.ac.uk.

End of Block: endofsurvey

**Supplemental 4** - **
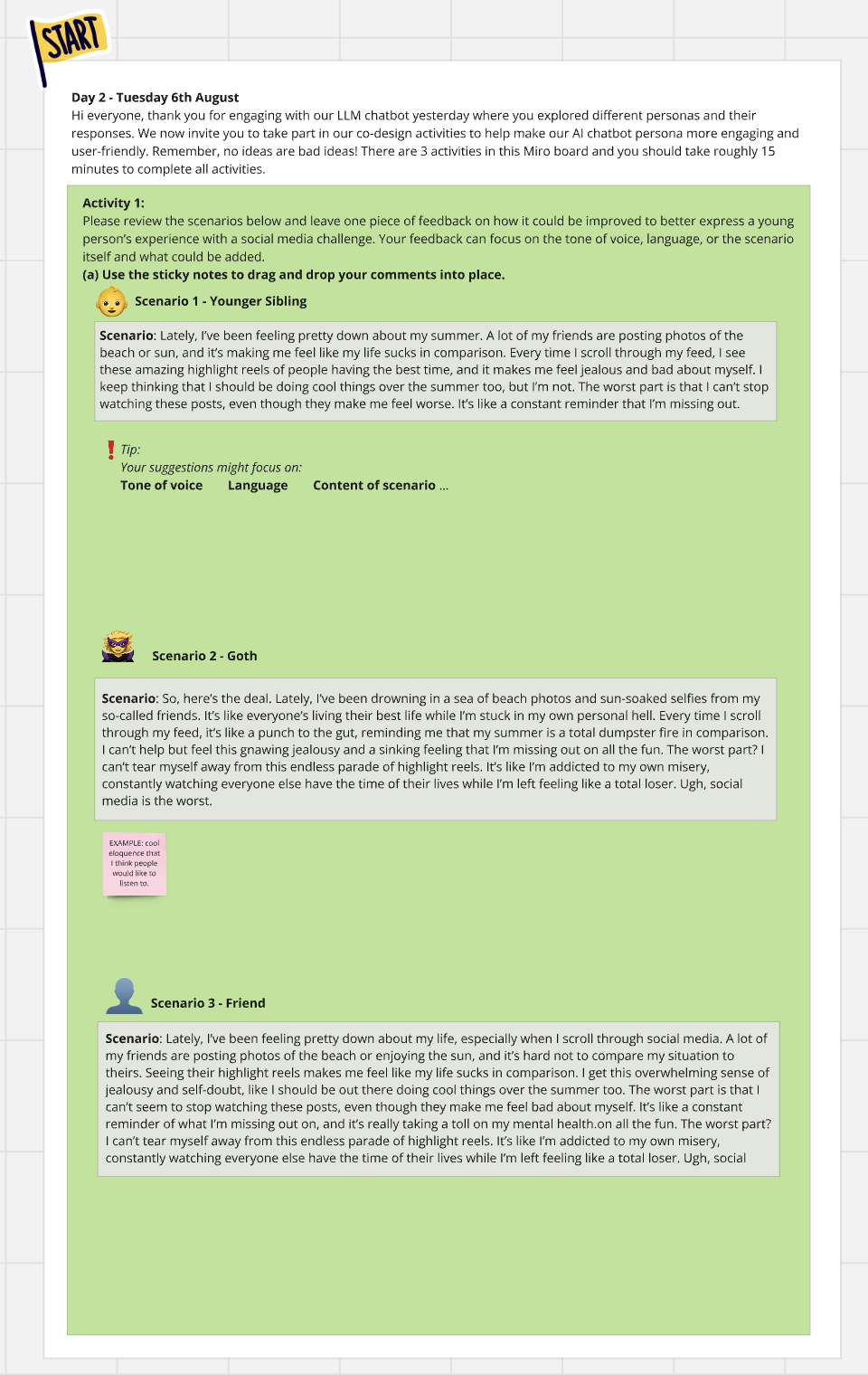
Activity 2 - Finding the Right Chatbot Voice**


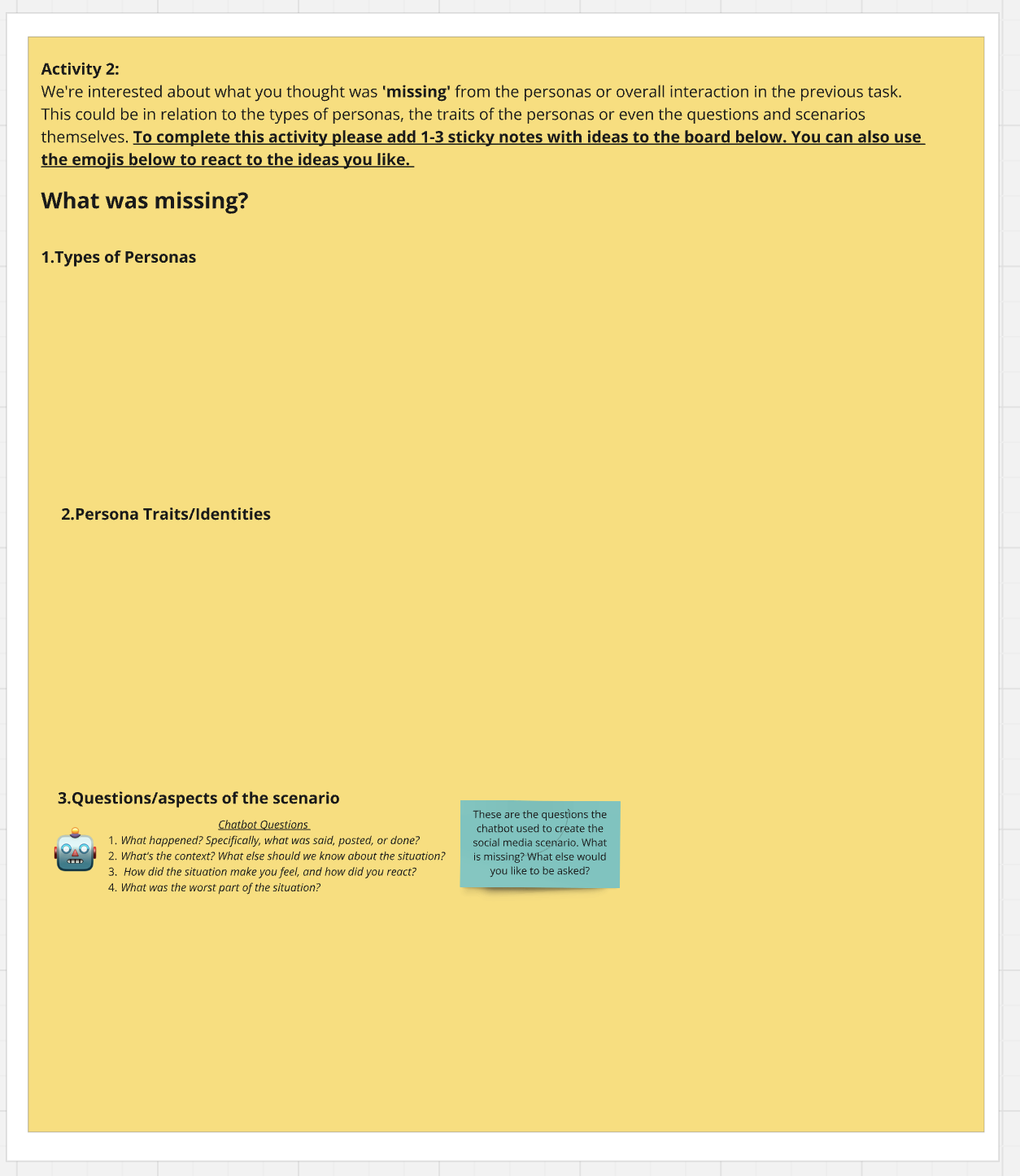

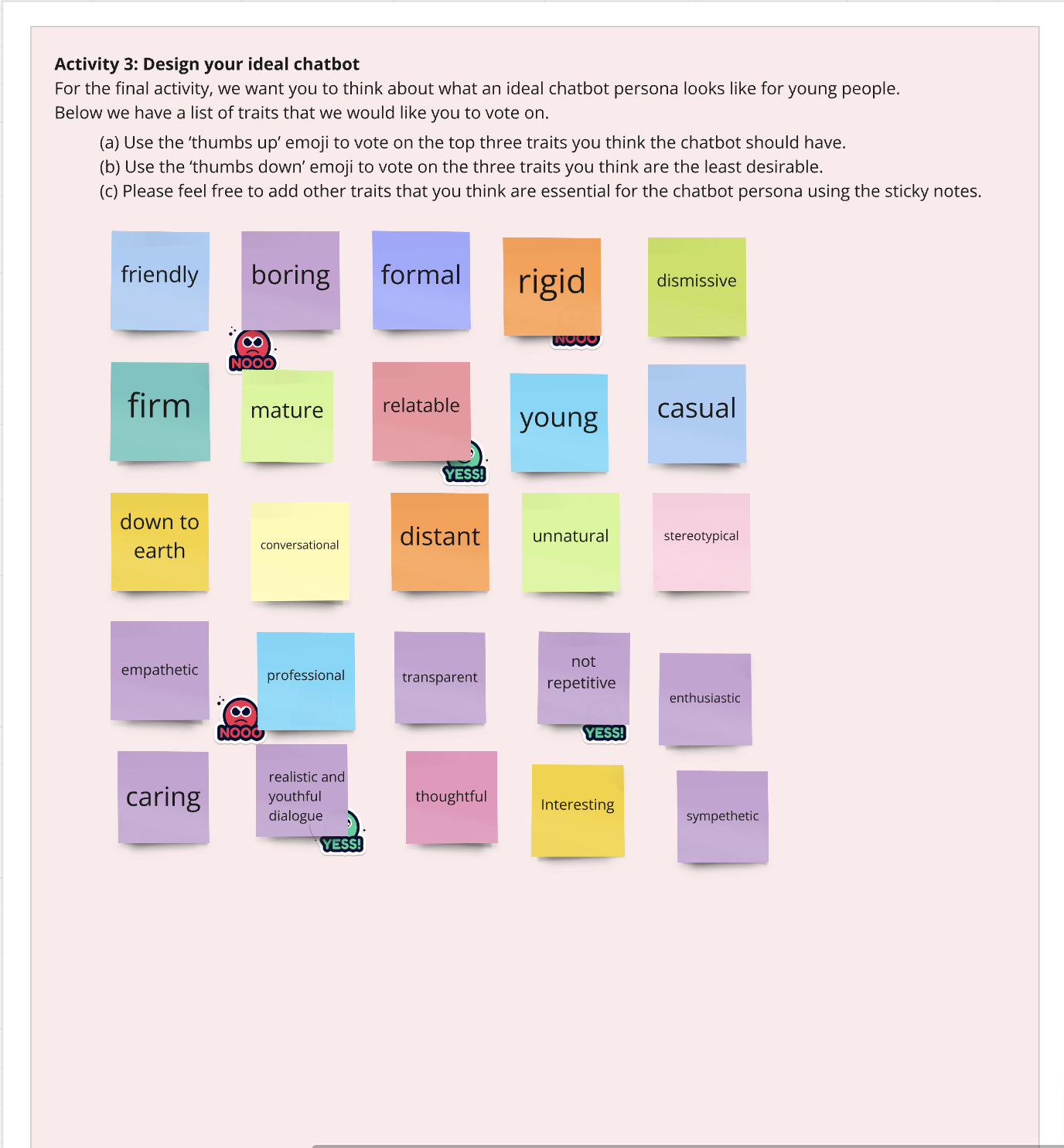


**Activity 4 - Looking Further: Exploring Next Steps and Applications**


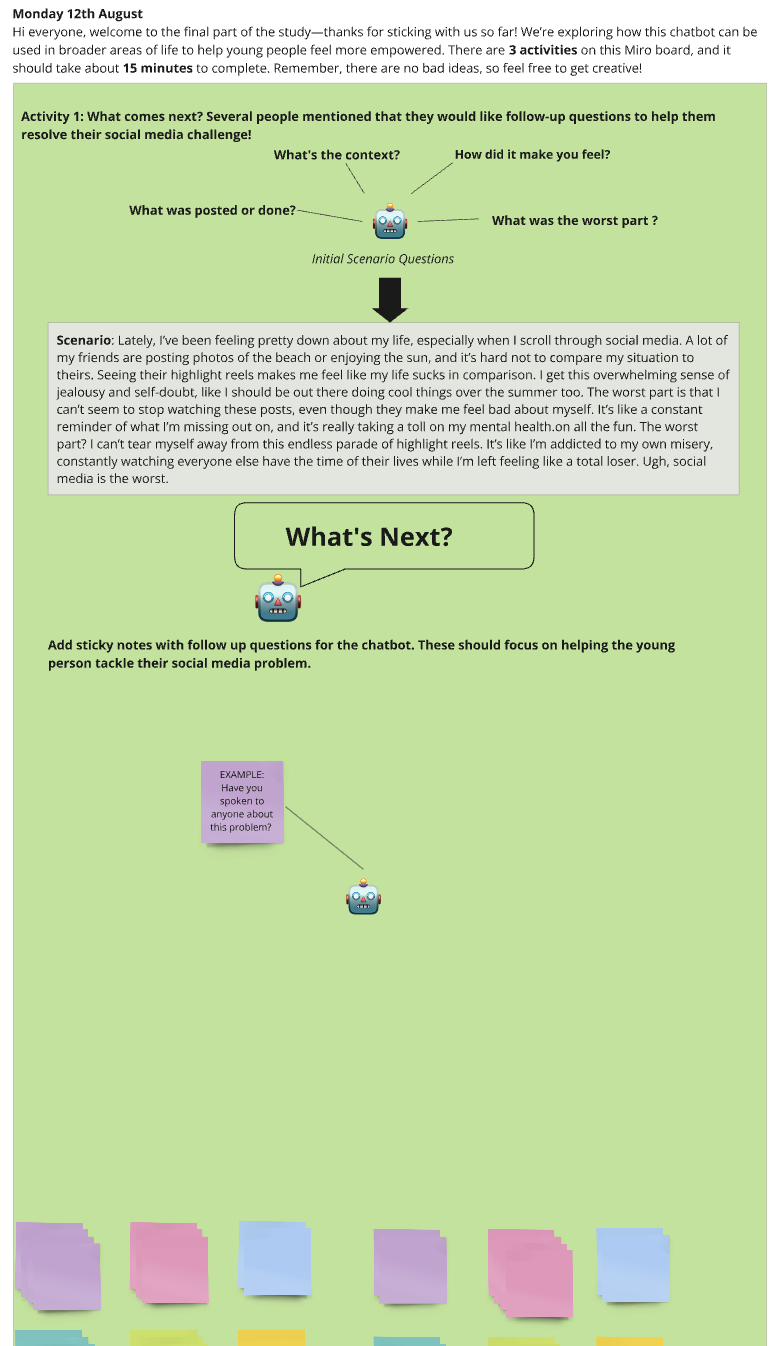


**
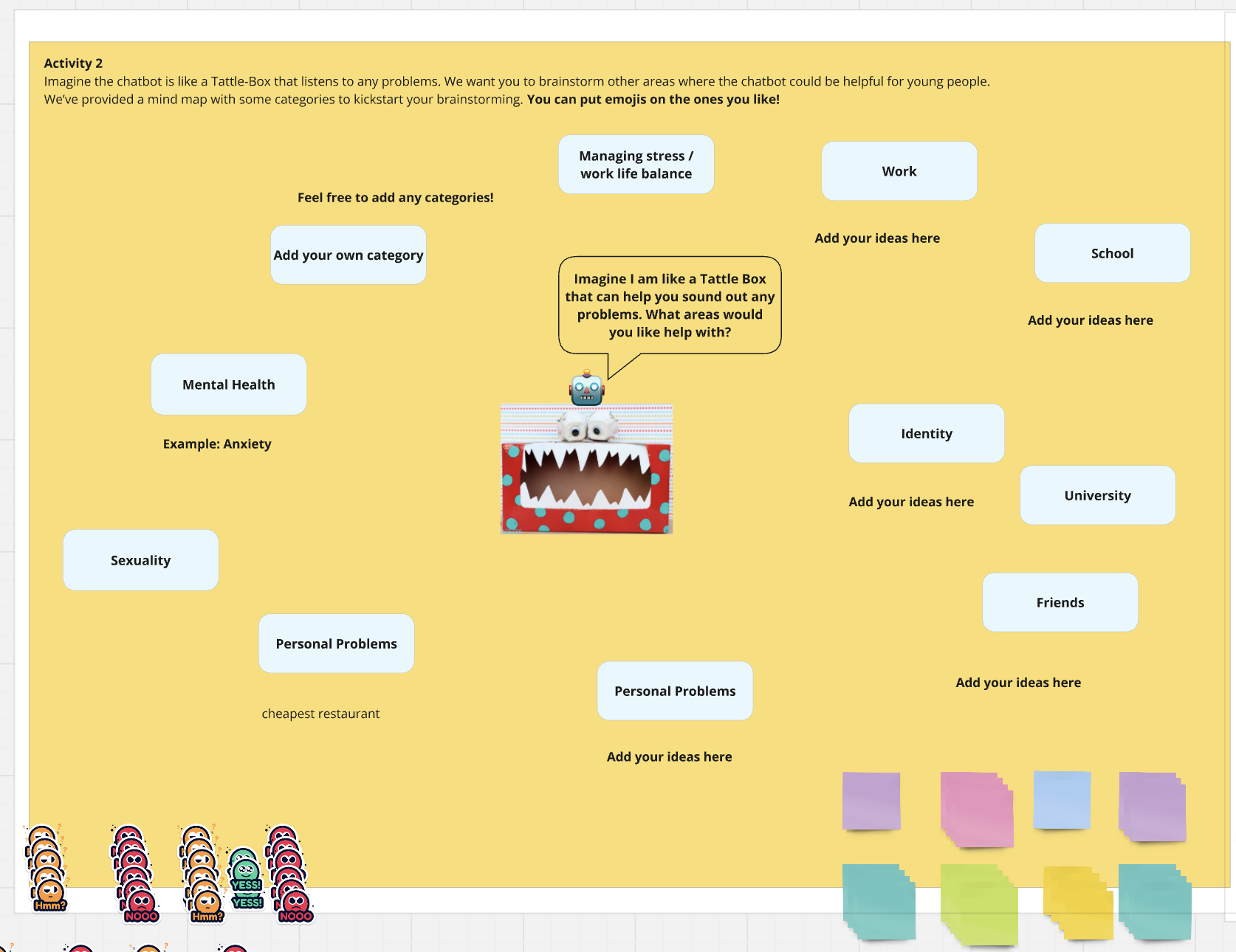
**


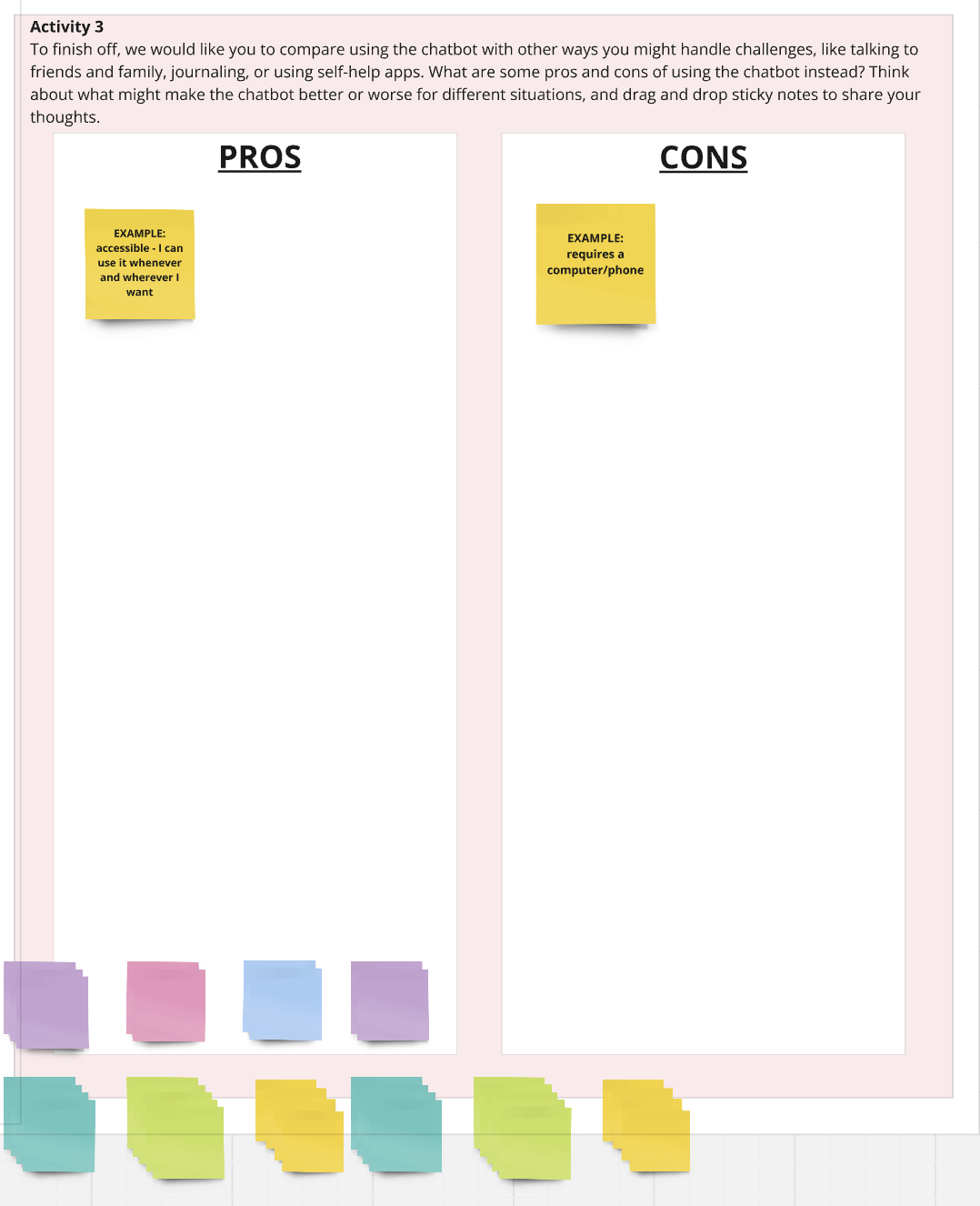


**Supplemental 5 - Interview Guides ARC – Semi-Structured**

- *Researcher Introduction*
- *To start with, I’m going to show you an overview of the chatbot interaction to remind you of the interaction flow.*

**Topic 1: Articulation**

**To start off with, what were your overall thoughts about the interaction with the chatbot?**

**We are interested in how the chatbot helps young people articulate or communicate their experiences around social media. Having recently interacted with the chatbot, how helpful or unhelpful did you find it to help you express social media experiences?**

**Were there any parts of the interaction that you found particularly helpful or unhelpful/annoying? This could include the types of questions that were asked, the length or content of the scenarios or the scenario feedback process.**

**We’re interested in how our chatbot interaction compares to other ways in which you’ve reflected on social media experiences. For example, talking to your friends or parents about social media challenges. How did the chatbot feel different to these conversations? Was there anything you particularly liked/disliked about the chatbot process in comparison?**

**Topic 2: Application**

**Thank you, now I want to briefly chat about other areas for which this kind of prototype or interaction might be useful. In this study, we asked young people to explore social media challenges, however, the chatbot can be adapted and could ask young people about a range of other areas.**

**Do you have any ideas or thoughts about other areas where this kind of bot might be useful for young people?**

**Are there any areas that you might like to apply this chatbot in your daily life? E.g., are there any things you find you struggle to articulate or talk about….**

*Thank you so much for taking part. It’s been really useful to hear your thoughts and opinions on the study and thank you for taking part in the initial steps of this study.*

*Is there anything you would like to ask me before we finish?*

**Supplemental 6 – Detailed Persona Feedback**

**ARC Activity 1 and 2 Persona Feedback**

| **Persona** | **Personas** | **Feedback (**+/–) |
| --- | --- | --- |
| **Friend** | You're a 23-year-old who is collecting stories of difficult experiences that your friends have on social media. You're trying to use the same tone and language as your friend has done, but you can reframe what they are saying a little to make it more understandable to others. | - *“It felt the most down-to-earth and realistic. It felt like something a friend would genuinely confide in you to discuss.”* - *“Whilst it was really good, it may have come across a bit too formally. However, this could be fine still as being too informal comes across as cringe.”* |
| **Younger Sibling** | You're a 14-year-old teenager who is collecting stories of difficult experiences that your friends have on social media. Use a language that you assume the friend would use themselves, based on their response. Be empathic, but remain descriptive. | - *“It was the most accurate that the chatbot presented to me.”* - *“Seemed too formal, didn't speak with enough slang to emulate a 14-year-old.”* |
| **Influencer** | You're a 25-year-old social media influencer who is collecting stories of difficult experiences that your followers have on social media. Use a language that is trendy and engaging, as you would on your social media platforms. Be empathetic, but remain descriptive and relatable. | - *“It was most accurate to how I think the influencers I consume on social media would speak.”* - *“It seemed very superficial. It also seemed stereotypical. I think the problem was the assumption that an influencer must be snobby.”* |
| **Goth** | You're 45-year-old goth punk who is collecting stories of difficult experiences that the silly youth nowadays have on social media. Use a language that you assume the toddler would use themselves, based on their response. Be edgy and cheeky in your response but remain marginally respectful. | - *“It was less robotic and more human as opposed to the others, I could imagine the person more clearly in the text.”* - *“I did not like the tone of voice used to convey the message, which almost turned the situation into a joke.”* |
| **Psychologist** | You're an expert developmental psychologist who is collecting stories of difficult experiences that your clients have on social media. Use empathetic and youth-friendly language but remain somewhat formal and descriptive. | - *“I think it came across formally but in a non-patronising way. It seemed like an adult you would want to listen to and respect their advice.”* - *“All it did to emulate this persona was elevate the literacy level and vocabulary used which I feel does not really present a psychologist persona but rather someone of a higher education and/or intellect.”* |

**Supplemental 7 – Tables**

Table 1 Demographics for User-Centred Pilot Studies

|  | **Pilot Study**  (n = 100) | **ARC Study**  (n = 30) |
| --- | --- | --- |
| **Age** |  |  |
|  | 18-20 | 18-20 |
| **Gender** |  |  |
| Male | 53 | 18 |
| Female | 46 | 12 |
| Prefer not to say | 1 |  |
| **Ethnicity** |  |  |
| White | 56 | 9 |
| Asian | 32 | 15 |
| Black | 6 | 4 |
| Mixed | 4 | 2 |
| Unspecified | 1 |  |
| **Employment Status** |  |  |
| Fulltime | 5 | 2 |
| Part-time | 32 | 6 |
| Unemployed/non-paid work | 32 | 15 |
| Unspecified/due to start work | 31 | 7 |

Table 2 Summary of ARC Activities

| **Broader Aim** | **#** | **n** | **Name** | **Activity** | **Time** | **Flow** | **Format** |
| --- | --- | --- | --- | --- | --- | --- | --- |
| *To further test and finetune the interaction and understand the type of voice/persona the prototype should have to effectively engage participants* | **A1** | 30 | Trying Out Different Storytelling Voices | Using an internet browser, participants interacted with a web-based LLM chatbot to explore hypothetical social media experiences. They were given the opportunity to explore different personas  (e.g., younger sibling, friend) and choose the personas they preferred to articulate their experiences. Following the interaction, they provided feedback via a Qualtrics survey. | 25 minutes | Days 1-2 | Streamlit (LLM Chatbot Interaction) & Qualtrics Survey |
|  | **A2** | 29 | Finding the Right Chatbot Voice | Participants accessed a web-based group Miro board to engage in short tasks alongside other participants (n = 15 per group). They provided feedback on several personas explored in Activity 1. They were also asked to reflect on the chatbot’s tone, language and personality and design an ideal chatbot persona for young people. | 15 minutes | Days 4-5 | Miro Board |
| *To understand the extent to which the micronarrative process empowers the articulation of certain experiences for young people and the risks and benefits of this process* | **A3** | 10 | Diving Deeper:  Interviews | Participants participated in 30-minute, one-on-one interview with a researcher on Zoom. The interviews probed individual feedback and experiences of the prototype, the process of articulating social media experiences and further applications of the chatbot. | 30 minutes | Days 7-14 | Zoom Interview |
|  | **A4** | 18 | Looking Further: Exploring Next Steps and Applications | Participants accessed a web-based group Miro board to engage in short tasks alongside other participants (n = 15 per group). They were asked to brainstorm additional features that could be added to the chatbot to make it more helpful. They also reflected on the pros on cons of using chatbots and explored further potential applications for young people. | 15 minutes | Days 9-10 | Miro Board |

Table 3 Wording of the survey questions as seen by participants.

| **Question Group** | **Question Text** |
| --- | --- |
| Implicit comparison (between-subject) | Overall, how helpful was the [chatbot/Qualtrics form] in helping you articulate events that happened on social media? |
|  | Overall, how difficult was it to respond to the question [posed by the chatbot/in the Qualtrics form] on the previous page? |
|  | Overall, how valuable do you think it would be for other young people to participate in this kind of [chatbot/Qualtrics form] interaction? |
|  | How well does the final narrative [produced by the chatbot/you entered into the Qualtrics form] accurately capture the objective circumstances of the situation (i.e., what factually happened in the situation, as opposed to your feelings about it)? |
|  | How well does the narrative [generated by the chatbot/you entered into the Qualtrics form] accurately capture how you felt in the situation? |
|  | If someone else who did not know anything about you or the situation read this narrative, how well would they understand your experience? [bot-generated narrative/Qualtrics form narrative] |
| Explicit comparison (within-subject) | Which process did you personally prefer: interacting with the bot to produce a final narrative or filling out the Qualtrics web form? |
|  | Which process felt easier to capture what you wanted to share about your experience? |
|  | Which process would be more appropriate when asking young people to share their stories? |
|  | Which process was more helpful for allowing you to make sense of your experience? |
|  | Which process did you feel better captured your voice? |
|  | Which process was more likely to make you consider how you can address your social media challenge? |
|  | How likely would you be to recommend each one of these experiences to a friend? [bot/Qualtrics form] |
|  | Did you have any privacy concerns about the [bot/Qualtrics form]? |
